# Meta-analyses of individual versus group interventions for pre-school children with autism spectrum disorder (ASD)

**DOI:** 10.1371/journal.pone.0196272

**Published:** 2018-05-15

**Authors:** Yoshiyuki Tachibana, Celine Miyazaki, Masashi Mikami, Erika Ota, Rintaro Mori, Yeonhee Hwang, Akiko Terasaka, Eriko Kobayashi, Yoko Kamio

**Affiliations:** 1 Division of Infant and Toddler Mental Health, Department of Psychosocial Medicine, National Centre for Child Health and Development, Tokyo, Japan; 2 Smart Aging International Research Center, IDAC, Tohoku University, Sendai, Japan; 3 Department of Child and Adolescent Mental Health, National Center for Neurology and Psychiatry, Tokyo, Japan; 4 Department of Health Policy, National Research Institute for Child Health and Development, Tokyo, Japan; 5 Department of Biostatistics, Clinical Research Centre, National Centre for Child Health and Development, Tokyo, Japan; 6 Department of Global Health Nursing, Graduate School of Nursing Science, St. Luke’s International University, Tokyo, Japan; 7 Department of Education, Tohoku Fukushi University, Sendai, Japan; 8 Department of Educational Collaboration, Osaka Kyoiku University, Osaka, Japan; TNO, NETHERLANDS

## Abstract

There is little evidence regarding the effects of individual and group intervention for children with autism spectrum disorder (ASD) on important outcomes. We performed meta-analyses using a random effects model to investigate the effectiveness of the individual and group intervention studies and to compare the effectiveness of these two types if possible. The main analysis which excluded studies at a high risk of bias (Analysis I) included 14 randomised controlled trials targeting children with ASD≤6 years of age (594 children). The results suggested that both individual and group interventions showed significant effects compared to the control condition on “reciprocity of social interaction towards others” (standard mean difference[SMD] [95%confidence interval{CI}] = 0.59[0.25, 0.93], p = 0.16; 0.45[0.02, 0.88], p = 0.39, respectively). Only individual interventions showed significant effects compared to the control condition on “parental synchrony” (SMD [95%CI] = 0.99 [0.70, 1.29], p<0.01). Our results showed no significant differences between individual and group interventions in effects on “autism general symptoms” (no study available for group intervention), “developmental quotient” (no study available for group intervention), “expressive language” (p = 0.56), “receptive language” (p = 0.29), “reciprocity of social interaction towards others” (p = 0.62), or “adaptive behaviour” (p = 0.43). We also performed sensitivity analyses including studies that had been excluded due to being at a high risk of potential bias (Analysis II). The results suggested that “reciprocity of social interactions towards others” showed significant effects for individual intervention compared to the control condition (0.50[0.31,0.69], p<0.001) but not for group intervention (0.23[-0.33, 0.78], p = 0.42). Individual intervention also showed significant effects on “parental synchrony” (0.98[0.30,1.66], p = 0.005) in the sensitivity analysis. The results also suggested no significant difference on all the outcomes between the individual and group interventions. We also reanalysed the data using cluster-robust standard errors as sensitivity analyses (Analysis III). Analysis III showed no significant effects in the intervention condition compared to the control condition on all the outcomes for both individual and group interventions. When Analysis II was reanalysed using cluster-robust standard errors (Analysis IV), individual interventions showed significant effects compared to the control condition on “reciprocity of social interaction towards others” and "parental synchrony" (mean estimate[95%CI], robust standard error, p = 0.50[0.20, 0.81], 0.13, 0.006; and 1.06[0.08, 2.05], 0.42, 0.04, respectively), and none of the outcomes showed significant effects under the intervention condition compared to the control condition for group interventions. The discrepancies in the results between the main analysis (Analysis I) and the sensitivity analyses (Analyses II, III, and IV) may be due to the small number of included studies. Since the outcome of “reciprocity of social interaction towards others” can be a dependent variable that is usually measured in a context-bound setting with the child's parent, we cannot conclude that individual interventions for pre-school children with ASD have significant effects on generalised skills for engaging in reciprocal interactions with others, even if the interventions have significant effects on the outcome. However, the outcomes of “reciprocity of social interaction towards others” may be promising targets for both individual and group interventions involving pre-school children with ASD. “Parental synchrony” may also be a promising target for individual interventions.

**Trial registration:** (CRD42011001349).

## Introduction

Many individual intervention programmes for pre-school children with ASD have been developed [1, 2]. Group intervention programmes are also common [3]. However, there is little rigorous evidence regarding the differences in the effects of individual and group interventions on important outcomes.

Individual and group intervention programmes have different characteristics, and both have their own strengths. One of the major differences between the two types of interventions is that individual intervention is delivered in a one-on-one environment, and group intervention is delivered as a uniform programme for a group. The one-on-one environment may be a great advantage for individual interventions compared to group interventions, as individual intervention can be tailored to each child, matching the developmental stage with the needs of the child and parents. In addition, in individual interventions, the child has more opportunities to talk to the therapist than in group interventions, which may be beneficial for enhancing children’s expressive language. Some individual interventions have a high intensity in terms of the frequency and time compared to group lessons (e.g. 40 hours per week for several years) [4], and such high-intensity individual therapy can be costly. One of the main advantages of group interventions is that this approach can provide children with the opportunity to engage with other children in a group setting. Group interventions allow children to learn the group’s rules and develop social skills [5]. Furthermore, parents can meet other parents at these group sessions, which can lead to peer support and information sharing [6]. Group interventions can provide opportunities for children to learn adaptive behaviours by participating in group activities [7]. Since group intervention can provide multiple children with a uniform programme at the same time, it may be more efficient and cost-effective than individual intervention.

Investigating how effective individual and group interventions are for achieving specific outcomes and comparing the effectiveness of the two intervention types will help identify the approaches that support effective intervention for children with ASD. The findings of these analyses will aid families, clinicians, and policymakers in determining which intervention programme they should adopt for children with ASD. The results will also help intervention developers understand the weak points of their interventions and add supplemental approaches to improve those weaknesses.

In this study, we categorised previously conducted randomised controlled trials (RCTs) of intervention programmes for children with ASD into two groups: individual and group interventions. The objective of our study was to investigate the effectiveness of the individual and group intervention studies and to compare the effectiveness of these two types if possible. We conducted a meta-analysis of methodologically adequate studies in accordance with the Cochrane Collaboration’s Systematic Reviews [8], which made it possible, for the first time, to report evidence to compare individual versus group interventions for pre-school children with autism spectrum disorder (ASD).

## Methods

**Fig 1:**
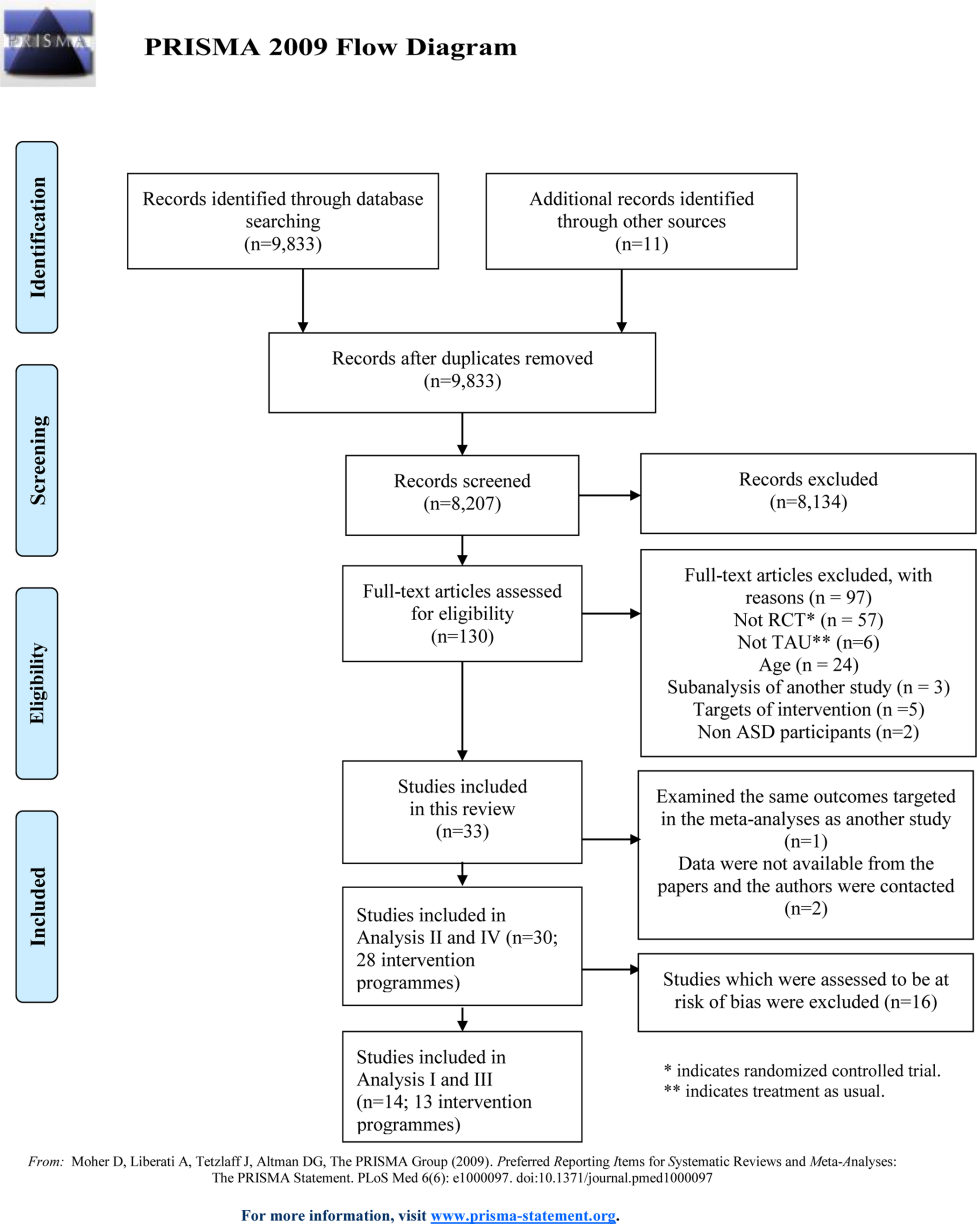
PRISMA 2009 flow diagram for the present meta-analysis study.

The methods used to conduct this study were in accordance with the Cochrane Handbook for Systematic Reviews [8]. The PRISMA guidelines [9, 10] were used to prepare this review’s protocol [11] (see Fig 1, S1 Appendix, and S7 Table).

### Selection criteria

#### Types of studies

We included RCTs, quasi-RCTs, and crossover trials.

#### Types of participants

Participants were children ≤6 years of age with a diagnosis of ASD, as below.

- Diagnostic and Statistical Manual of Mental Disorders Third Edition—Revised (DSM-III-R) [12], Diagnostic and Statistical Manual of Mental Disorders Fourth Edition (DSM-IV) [13], Diagnostic and Statistical Manual of Mental Disorders-IV-Text Revision (DSM-IV-TR) [14]: autistic disorder, Asperger disorder, pervasive developmental disorder not otherwise specified (PDD-NOS)
- International Classification of Diseases-10 (ICD-10) [15]: childhood autism, atypical autism, Asperger syndrome, other pervasive developmental disorders, pervasive developmental disorders, unspecified.
- Diagnostic and Statistical Manual of Mental Disorders-5 (DSM-5)[16]: autism spectrum disorder

#### Types of interventions

Two types of interventions were targeted in this study: individual and group interventions. Individual interventions were defined as those consisting of individual sessions in which the therapist interacts with the child on a one-on-one basis. Group interventions were defined as those consisting of group sessions without individual sessions. Studies with interventions delivered to the parents or guardians and/or directly to the child by psychologists, speech pathologists, special educators, teachers, or other allied health professional students were included. We classified the studies reviewed into these two types of interventions and limited the analysis to pre-school children ≤6 years of age. Studies describing pharmacological, alternative, or complementary medicine interventions were excluded. Studies in which the control group received a specific intervention that was not considered “treatment as usual” for children with ASD provided by their local services were excluded.

#### Types of outcomes

We systematically assessed the various outcomes used within recent intervention programmes. Among many outcomes, we prioritised autism symptoms as the primary outcome (see I below). In addition, we investigated other relevant secondary outcomes (see II below), which are important for children’s daily lives and prognoses. We chose outcomes that had been investigated in previous studies [17–23].

I. Primary outcomes. 1.1. Autism general symptoms. This outcome indicated the severity of autism symptoms related to the defining symptoms of autistic disorder in DSM-IV-TR, based on the score of the Autism Diagnostic Observation Schedule (ADOS) [24, 25].

II. Secondary outcomes. Outcomes related to children’s developmental level, which are not required for an autism diagnosis, were used for the analyses. We defined the “developmental quotient” as a combination of the developmental quotient and intelligence quotient. In this paper, we reported five outcomes, as below. “Reciprocity of social interaction towards others” was defined as success in reciprocal social interactions with his/her parents or examiners and joint engagement for communication with others. We used the included studies’ data for this outcome when the outcome was measured by a communication measurement tool rather than an autism-specific measurement tool. 2.1. Developmental quotient. 2.2. Expressive language. 2.3. Receptive language. 2.4. Reciprocity of social interaction towards others. 2.5. Adaptive behaviour.

We also reported other outcomes described below in the Appendices. “Qualitative impairment in social interaction” (3.1), “Qualitative impairment in communication” (3.2), “Restricted repetitive and stereotyped patterns of behaviour, interests, and activities” (3.3) were the triad of the diagnostic criteria of ASD in DSM-IV-TR [26]. In this study, we used the included studies’ data for these outcomes only when they had obtained them using ASD-specific measurement. We also clarified “Parental synchrony” (3.6) as parental sensitive responding, non-directive, and emotional attunement to the child via verbal and non-verbal interactions. 3.1. Qualitative impairment in social interaction. 3.2. Qualitative impairment in communication. 3.3. Restricted repetitive and stereotyped patterns of behaviour, interests, and activities. 3.4. Initiating joint attention. 3.5. Responding to joint attention. 3.6. Parental synchrony. 3.7. Parenting stress.

Regarding autism symptom outcomes 1.1, 3.1, 3.2, and 3.3, studies without objective coding of child observation (e.g. only using a parent questionnaire or parent interview) were excluded from the analyses.

### Electronic search (see S2 Appendix)

We searched the following databases: PsycINFO, Medline via Ovid, ERIC, CINHAL, and the Cochrane Central Register of Controlled Trials (CENTRAL) without any language restriction on October 2, 2014.

We used the following search terms to search all trial registries and databases: “autism,” “autism spectrum disorder,” “ASD,” “high function autism,” “high function ASD,” “Asperger syndrome,” “pervasive developmental disorder,” “PDDNOS,” “intervention,” “treatment,” “therapy,” “communication,” “interpersonal,” “speech,” “interaction,” “synchrony,” “relationship,” “language,” “social,” “development,” “behaviour,” “intensive behavioural intervention,” “trial,” and “outcome.” The search was limited by children’s age (only those studies including children 0–6 years of age) and study type (“randomised controlled trial”). The search strategy was reviewed by the librarians of the National Research Centre for Child Health and Development and the University of Manchester. Other relevant studies were also searched for using reference lists to identify trials and review articles.

### Search of other resources

References from identified trials and review articles were manually searched to identify any other relevant RCTs. ClinicalTrials.gov and CENTRAL were also searched for RCTs that were registered as completed but not yet published.

### Data collection and analyses

All references found using this search strategy were compiled using the reference-management software programme EndNote X6 (Thomson Reuters, New York City, NY, USA). Two authors (AT and EK) independently reviewed the abstracts of potentially relevant studies. EK initially screened the titles and abstracts and eliminated all citations that were not relevant to this study. Thereafter, two of the five review authors (YT, YH, EK, CM, and AT) assessed. If there was any disagreement between the assessors concerning the inclusion of a study, a third referee (EK or YT) discussed the matter with them and reached a resolution by inspecting the paper. Final inclusion of articles for the meta-analyses was judged by YT and EK, and supervised by OE based on the results of the risk-of-bias assessments.

### Data extraction and management

EK and CM independently extracted data from the selected studies using a data-extraction form. The extracted data consisted of the diagnosis, country, number of participants, children’s ages, name of the intervention programme, type of intervention, methods (dose and frequency of intervention), characteristics of the control group, and duration. If there were disagreements regarding data extraction, they were resolved by discussing with a third author (YT).

### Assessment of risk of bias

Five independent review authors (YT, YH, EK, AT, and CM) assessed the risk of bias. If there were disagreements concerning those assessments, they were resolved by discussion with a referee (either YT or EK). We used the Cochrane Collaboration’s assessment tool of risk of bias for each included study [27]. The tool included the following domains: sequence generation, allocation concealment, blinding of participants and personnel, blinding of outcome assessment, complete outcome data, selective outcome reporting, and other sources of bias. The assessors evaluated whether or not there was a risk of bias in those domains. The potential risk of bias for each domain of each study was judged as follows: “low risk of bias,” “unclear risk of bias,” and “high risk of bias.” Whether or not a study should be included in the meta-analyses was judged individually based on the results of the risk of bias assessment. Studies judged to be at a high risk of bias were excluded.

### Assessment of reporting biases

When there was a sufficient number of studies (≥10), funnel plots were drawn to detect the distribution of the studies by their effect and sample sizes. Such a relationship could be the result of publication or related biases, or due to systematic differences between small and large studies. Every attempt was made to obtain unpublished data and data from conference proceedings.

### Management of missing data

For each study, missing data were handled as follows: When there was a significant quantity of participant data missing in the report, such that we agreed that the conclusions of the study were compromised, the trial authors were contacted. If a reply could not be obtained or the full data were not made available, those studies were excluded from the final analysis. We reported the reasons for missing data provided by the authors of the included studies. The extent to which the results of the review might be altered by the missing data were assessed using sensitivity analyses, which included the studies that had been excluded based on the exclusion criteria.

### Data synthesis

YT analysed the data using the Review Manager software programme, version 5.3 (Cochrane Collaboration Software, Copenhagen). We assessed continuous data, which were analysed whenever the mean values and standard deviations (SDs) were available and there was no clear evidence of a skew in the distribution. Meta-analyses were performed if two or more studies suitable for inclusion were found and considered to be satisfactory. To compare the two types of interventions (i.e. group versus individual), we categorised the studies into subgroups based on the two intervention models described above. Since the reviewed studies measured several outcomes in a nonuniform manner, outcome data were synthesised using the standardised mean difference (SMD; mean divided by SD post-intervention) for both intervention and control groups. Syntheses were based on previous meta-analyses of interventions for children with ASD [1, 2]. We synthesised the various categories of outcome measures using the SMDs for both groups. We tested the two subgroup interactions (i.e. individual versus group interventions) using a random effects model [27]. We used the I^2^ statistic to assess the rationale of data synthesis based on the degree to which there was heterogeneity in the types of measurement in the included studies [27]. We also performed an overall synthesis of the included studies for the two intervention types on each outcome. We analysed these studies in the same way in which previous meta-analyses amalgamated studies of children with ASD that had different intervention modalities and different measurements [1, 2]. MM and YT performed sensitivity analyses using cluster-robust standard error.

#### Main analysis

Analysis I (analysis excluding studies at a high risk of bias using a random effects model). We performed data syntheses after excluding studies that were assessed as having a “high” or “unclear” risk of bias in both “random sequence generation (selection bias)” or “allocation concealment (selection bias)” and studies with a “high” risk of bias in “incomplete outcome data (attrition bias).” We also performed sensitivity analyses, as described below. To interpret the results of the present study, we prioritised Analysis I. If there was a discrepancy in the results of Analysis I and the sensitivity analyses, we carefully assessed the reasons for these discrepancies in our interpretation.

#### Sensitivity analyses

Analysis II (analysis of all the included studies using a random effects model). We performed a sensitivity analysis with all of the included studies to explore the extent to which studies excluded from Analysis I might have affected the results of the meta-analysis.

Analysis III and IV (sensitivity analysis for Analysis I and II, respectively, which used cluster-robust standard error). We also performed sensitivity analyses of the overall effects on the outcomes for Analysis I and II (Analysis III and IV, respectively). This was because many of the studies had multiple dependent variables that were analysed and were nonindependent of effect sizes. This affected the confidence interval around the summary effect sizes, which could result in Type I error rate inflation. To address this issue, we analysed the data using sensitivity analyses by fitting random effects models with cluster-robust standard errors by clusters of internally correlated effect estimates [28] using the SAS software programme, version 9.4 (SAS Institute Inc., Cary, NC, USA).

Sensitivity analysis excluding studies with significant baseline imbalances). If a study included in a meta-analysis had a significant baseline imbalance in a measured outcome between the intervention and control groups, we also performed a sensitivity analysis with that study excluded.

### “Summary of findings for the main outcomes” table (Table 1)

We created a “Summary of the findings for the main outcomes” table (Table 1), which shows the main outcomes of this review that may be important to parents, clinicians, and decision makers. We used the Grades of Recommendation, Assessment, Development and Evaluation (GRADE) system [29] to describe the quality of evidence and the strength of recommendation and used the GRADEpro software programme (McMaster University and Evidence Prime Inc., Hamilton, ON, Canada) [30] to construct the tables. We expressed the quality of evidence on a four-point adjectival scale (“high,” “moderate,” “low,” or “very low”). EO coded the GRADE scales for the main outcomes of this study.

**Table 1:** Summary of findings for main outcomes (Analysis I: Random effects model, 14 studies).

Outcomes	Illustrative comparative risks* (95% CI)		Relative effect	Number of Participants	Quality of evidence	Comments
			(95% CI)	(studies)	(GRADE)	
	Assumed risk	Corresponding risk				
	Control	Autism general symptoms				
**Autism general symptoms**	Mean “autism general symptoms” was 0	Mean “autism general symptoms” in the intervention groups was **0.31 SD lower** (−0.63–0.01 higher)	SMD −0.31 (−0.63–0.01)	227	⊕⊕⊕⊝	
				(3 studies)	**moderate**^2^	
**Developmental quotient**	Mean “developmental quotient” was **0**	Mean “developmental quotient” in the intervention groups was **0.31 SD higher** (−0.02–0.65 higher)	SMD 0.31 (−0.02–0.65)*	208*	⊕⊕⊕⊝	Results of sensitivity analysis excluding studies with a significant baseline imbalance
				(4 studies)	**moderate**^2^	
**Expressive language**	Mean “expressive language” was 0	Mean “expressive language” in the intervention groups was **0.11 SD higher** (−0.07–0.3 higher)	SMD 0.11 (−0.07–0.3)	457	⊕⊕⊕⊝	
				(8 studies)	**moderate**^1^	
**Receptive language**	Mean “receptive language” was 0	Mean “receptive language” in the intervention groups was **0.12 SD higher** (−0.11–0.34 higher)	SMD 0.12 (−0.11–0.34)	457	⊕⊕⊕⊝	
				(8 studies)	**moderate**^1^	
**Reciprocity of social interaction towards others**	Mean “reciprocity of social interaction towards others” was **0**	Mean “reciprocity of social interaction towards others” in the intervention group was **0.53 SD higher** (0.29 more–0.78 more)	SMD 0.53 (0.29–0.78)	380	⊕⊕⊕⊕	
				(8 studies)	**High**	
**Adaptive behaviour**	Mean “adaptive behaviour” was **0**	Mean “adaptive behaviour” in the intervention group was **−0.04 SD higher** (−0.23 more–0.15 more)	SMD −0.04 (−0.23–0.15)	414	⊕⊕⊕⊝	
				(7 studies)	**moderate**^1^	

**Interventions for pre-school children with autism spectrum disorder (ASD). Population:** Pre-school children aged 6 years or younger with a diagnosis of ASD. **Settings:** Australia, Canada, Japan, Norway, UK and USA. **Intervention:** Interventions for pre-school children with ASD

*The basis for the assumed risk (e.g. median control group risk across studies) is provided in footnotes. The corresponding risk (and its 95% CI) is based on the assumed risk in the comparison group and the relative effect of the intervention (and its 95% CI).

CI: confidence interval; GRADE: Grades of Recommendation, Assessment, Development and Evaluation; SMD: standard mean difference; SD: standard deviation.

GRADE Working Group grades of evidence

**High quality:** Further research is very unlikely to change our confidence in the estimate of effect.

**Moderate quality:** Further research is likely to have an important impact on our confidence in the estimate of effect and may change the estimate.

**Low quality:** Further research is very likely to have an important impact on our confidence in the estimate of effect and is likely to change the estimate.

**Very low quality:** We are very uncertain about the estimate.

^1^ Small sample size with wide CI crossing the line of no effect.

^2^ Estimate based on small sample size.

## Results

### Search results

A flow diagram of the search results is shown in Fig 1. We found 9,833 citations using the search strategy run in October 2014, of which 8207 were abstracts of potential interest. If clarification or further data were needed, the trial authors were contacted. We contacted the authors of 10 trials, and 4 replied with the relevant data. The examination of the abstracts and full text of reports and manual searching of articles and reference lists resulted in 96 studies being excluded for the following reasons (see S3 Appendix): 57 studies were not RCTs, 6 did not have “treatment as usual” control conditions, 24 did not match the inclusion criteria for age, 2 were sub-analyses of previous studies, 5 did not fit the 2 intervention types that were targeted in this review, and 2 included children with non-ASD disabilities. Thirty-three studies [31–63] were thus included in the review. Of these, four reported results of other analyses from the original studies ([42] from [43], [61] from [45], [57] from [56], and [53] from [52], respectively), and one [61] examined the same outcomes as the original paper [45]. Two studies [62, 63] were excluded from the meta-analyses because the data required for the analyses were not supplied by the papers or provided after inquiring with the author. One study [60] investigated the effectiveness of the two programmes compared to a comparison group. Thus, a total of 30 papers [31–60] representing 28 intervention programmes [31–41, 43–52, 54–56, 58–60] were included in the current meta-analyses.

### Description of included studies

See: Characteristics of included studies (S1 Table)

All studies were RCTs; the active controls or psychological placebo groups consisted of treatment as usual by local services for children with ASD.

#### Intervention content

Eleven included studies were categorised as individual interventions [31, 33–35, 37, 41, 44, 54, 55], and three were categorised as group interventions [38, 43, 64].

#### Control condition

Our meta-analyses limited the targets only to the studies whose control conditions were “treatment as usual”.

#### Risk of bias

**Fig 2:**
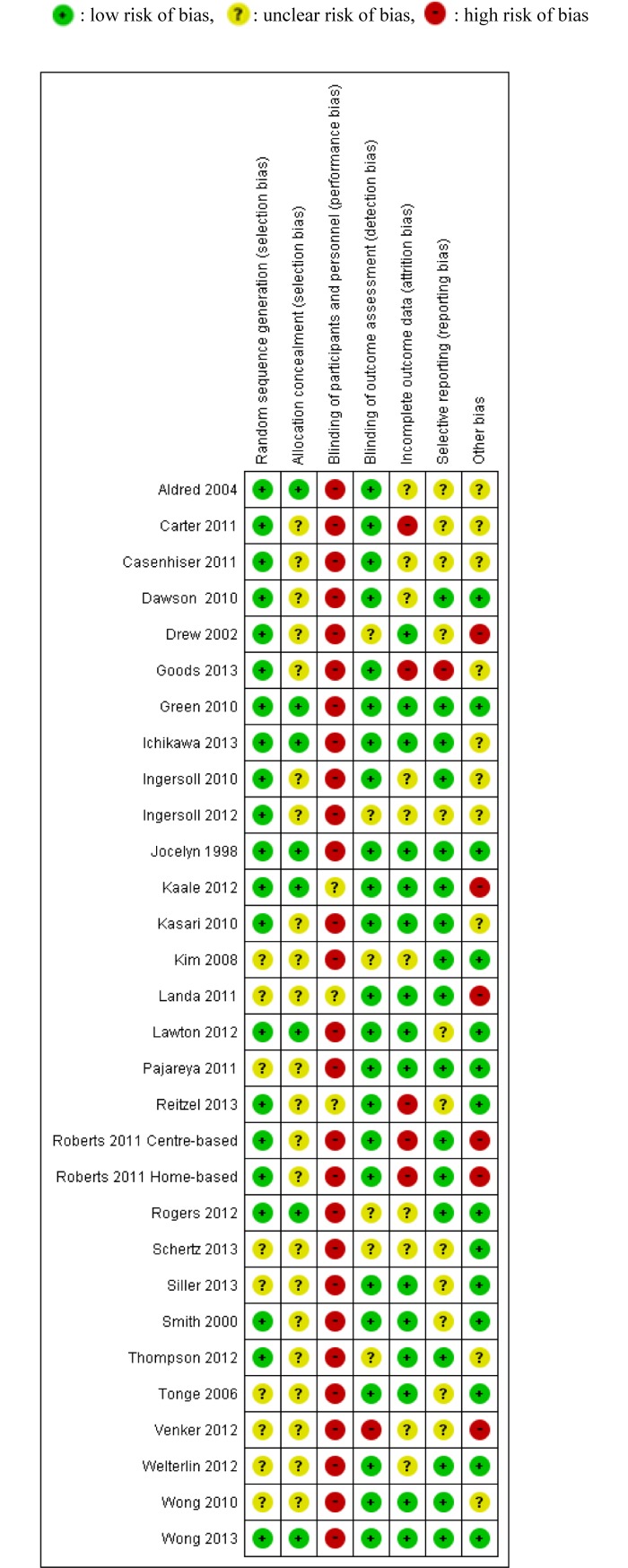
“Risk of bias” summary.

**Fig 3:**
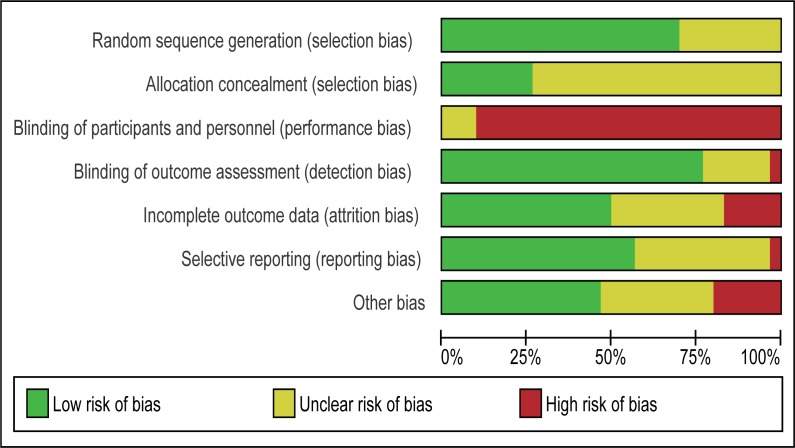
“Risk of bias” graph.

Summaries of “risk of bias” judgments are shown in Figs 2 and 3.

Summary. In summary, no studies were found to have a low risk of bias in all domains.

Random sequence generation. No trials were at a “high risk” of bias for sequence generation (not truly random); however, the method of randomisation was unclear (not reported) in two trials. One study did not mention the details of random sequence generation [65]. Another study [60] had three arms, and the two intervention arms were randomised by a computer, but the wait-list control groups were not allocated by random sequence generation.

Allocation (selection bias). Nine studies [31, 37, 38, 41–43, 47, 50, 62] mentioned allocation concealment, and the rest did not. Those that did not mention allocation concealment were rated as having an “unclear risk” of bias.

Blinding (performance bias). No studies were rated as having a “low risk” of bias; four studies [42, 43, 46, 49] were rated as having an “unclear risk” and the other 26 [12–14, 42, 43, 45–49, 51–60, 63, 65–69, 71] were rated as having a “high risk.” This was because, for interventions in this area, it was (by definition) impossible to blind parents and interveners from the intervention being performed.

Blinding (detection bias). Detection bias was not found to be a major influence in most included studies. One study was rated as having a “high risk” of bias [58]. In this study, given staffing constraints, observational coding and reliability coding were conducted by two graduate student clinicians; thus, it was impossible to maintain blinding to the treatment group assignment.

Incomplete outcome data (attrition bias). Twenty-six of the included studies addressed attrition in ways judged to be at “low risk” or “unclear risk” of bias. Three studies were rated as being at “high risk” of bias because of high attrition rates (more than 20%) [36, 49] and missing data [32].

Selective reporting (reporting bias). One study was rated as being at a “high risk” of bias because discussion of a measure was left out of the Discussion section of their report [36].

We regarded studies with a “high risk” of bias, which were excluded from quantitative data syntheses, as those with the following characteristics: (i) studies with an “unclear risk” of bias in both random sequence generation and allocation concealment and (ii) studies with a “high risk” of bias in incomplete outcome data. After assessing the risk of bias, 14 studies [31–44, 47, 49, 50, 54, 55, 64] were included in Analysis I (Figs 4–17), and 30 studies [31–60] were included in Analysis II.

**Fig 4:**
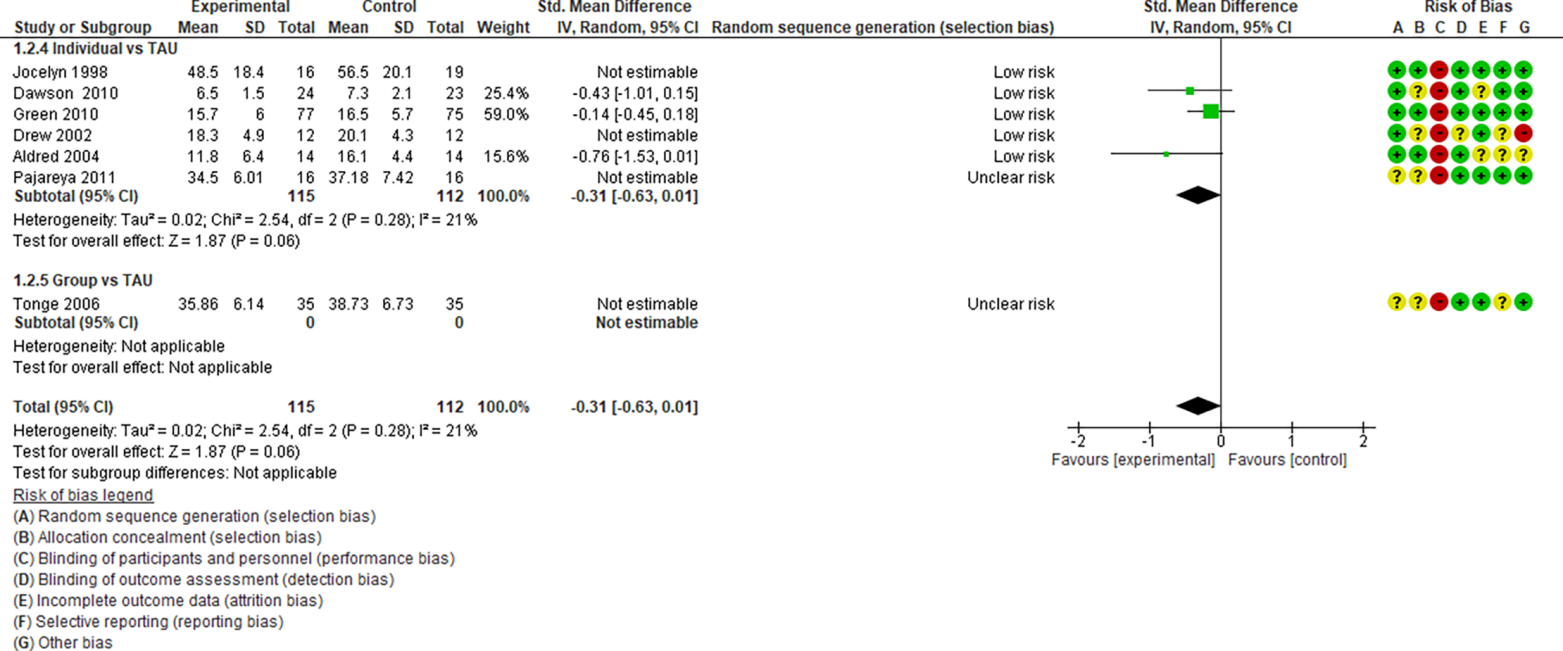
Forest plot of “autism general symptoms” (Analysis I).

**Fig 5:**
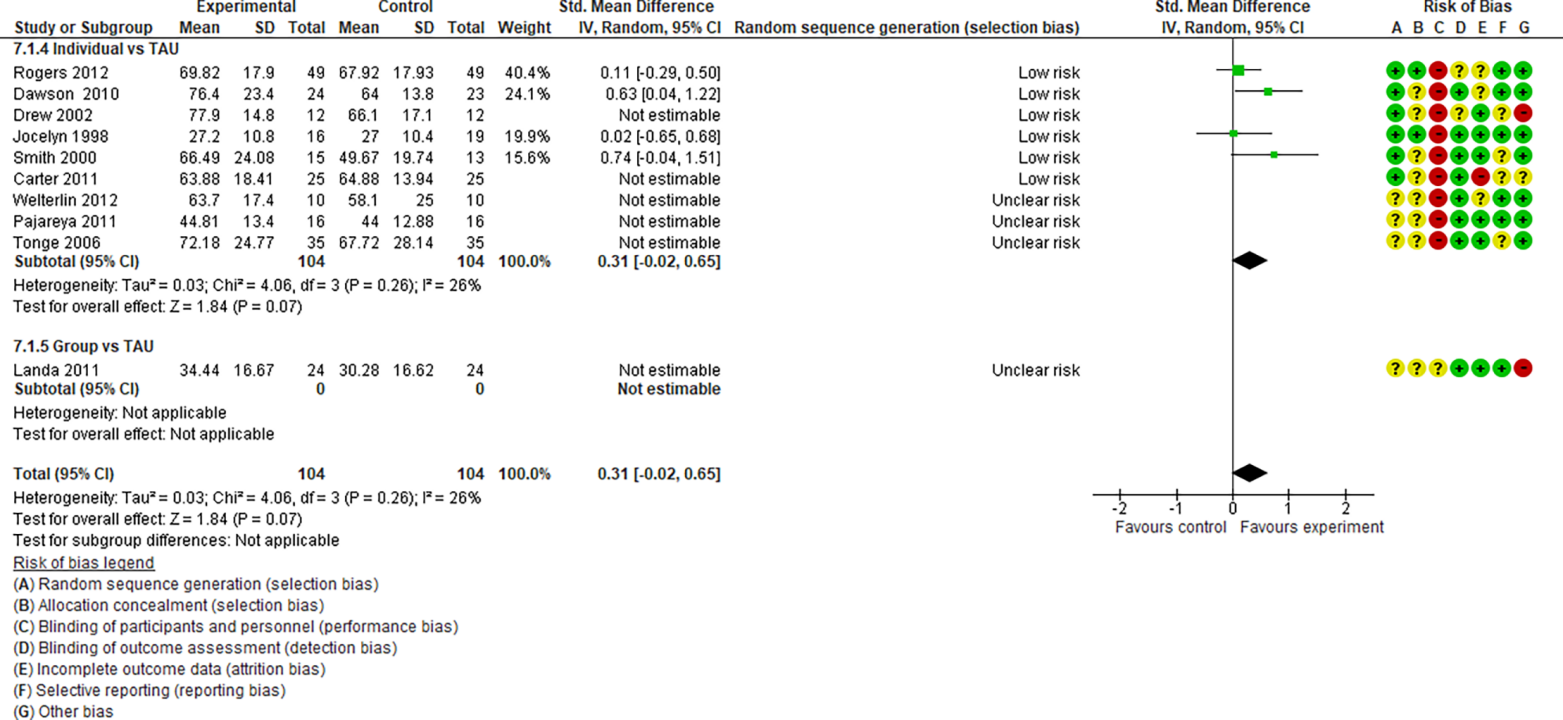
Forest plot of “developmental quotient” (Analysis I).

**Fig 6:**
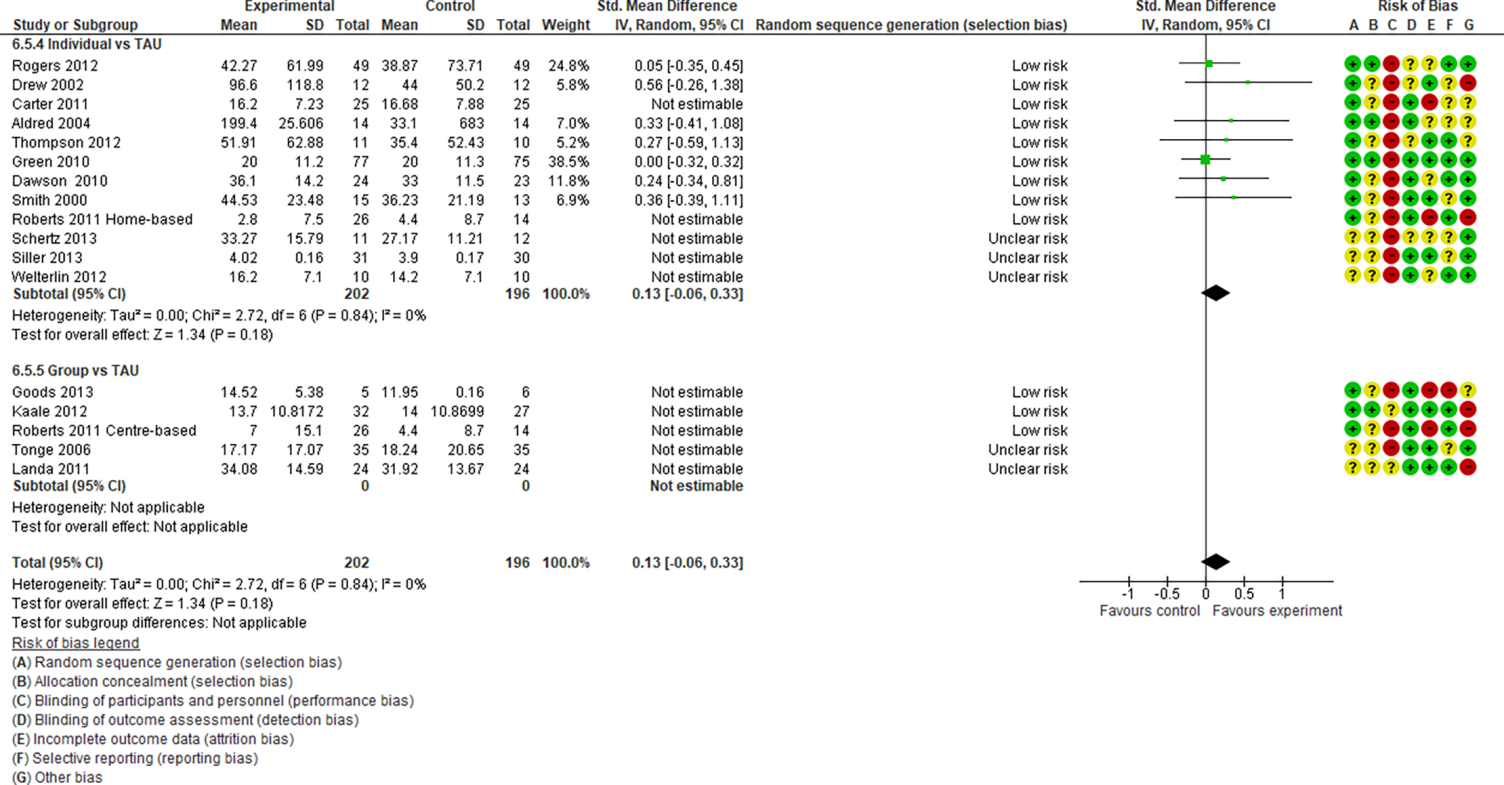
Forest plot of “expressive language” (Analysis I).

**Fig 7:**
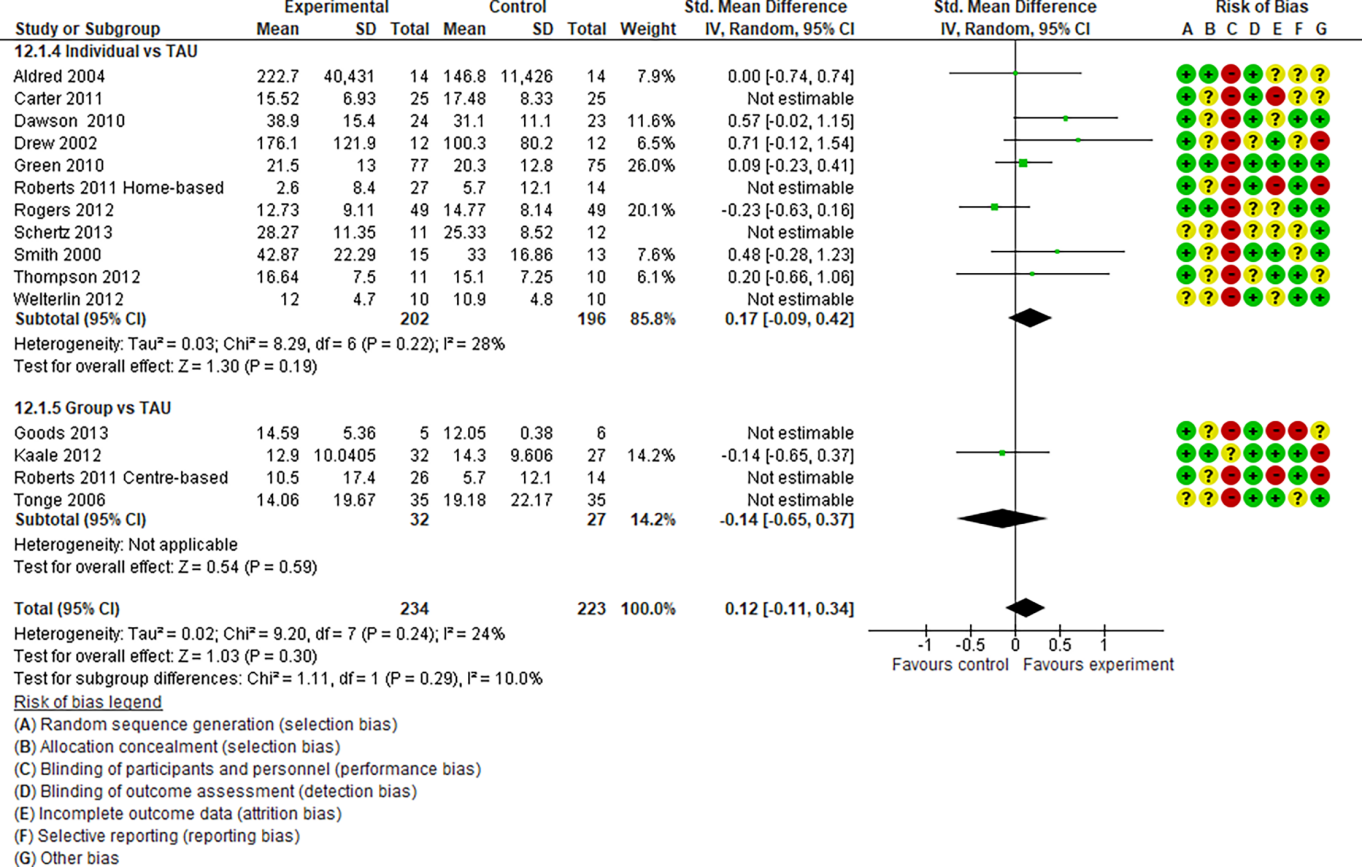
Forest plot of “receptive language” (Analysis I).

**Fig 8:**
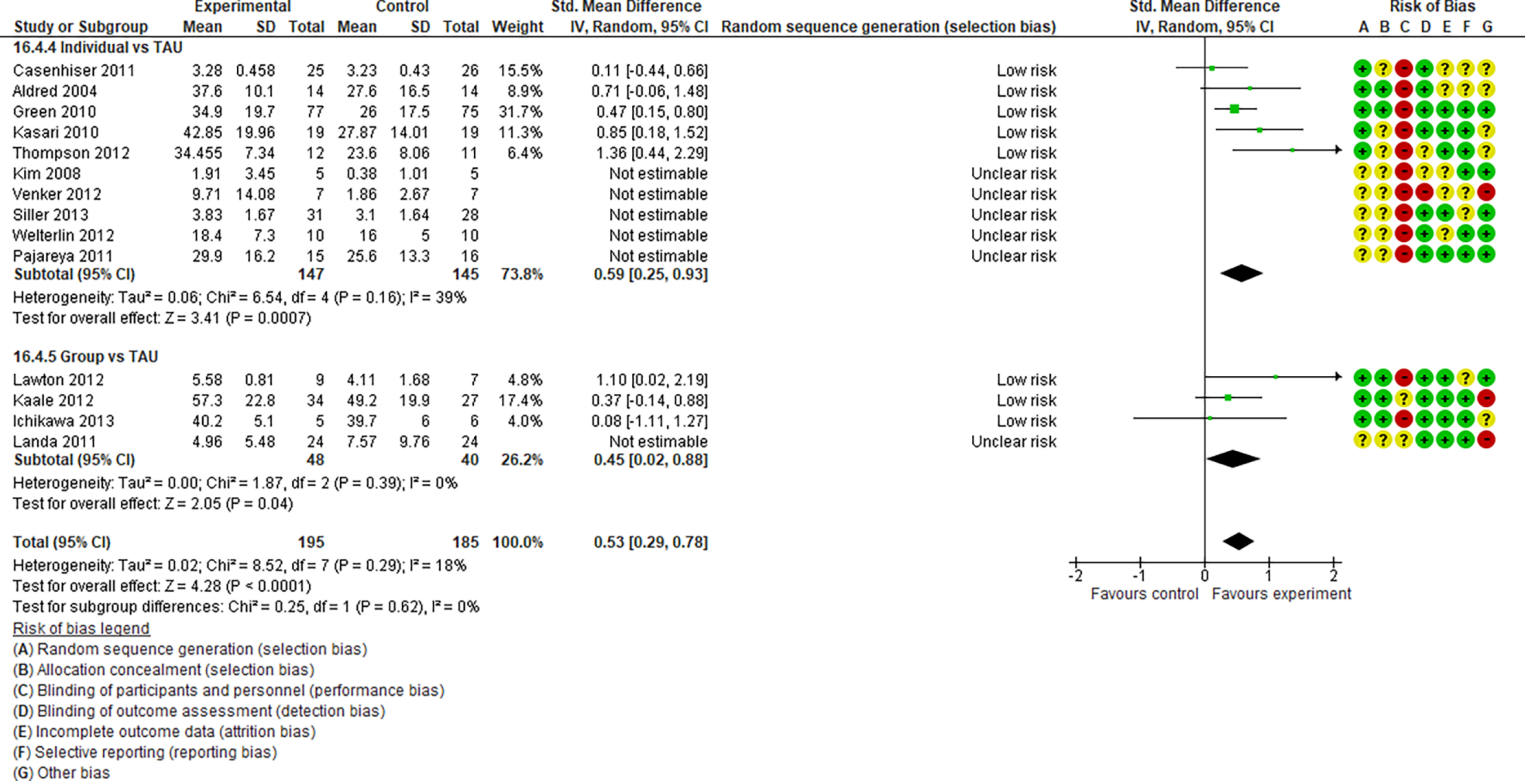
Forest plot of “reciprocity of social interaction towards others” (Analysis I).

**Fig 9:**
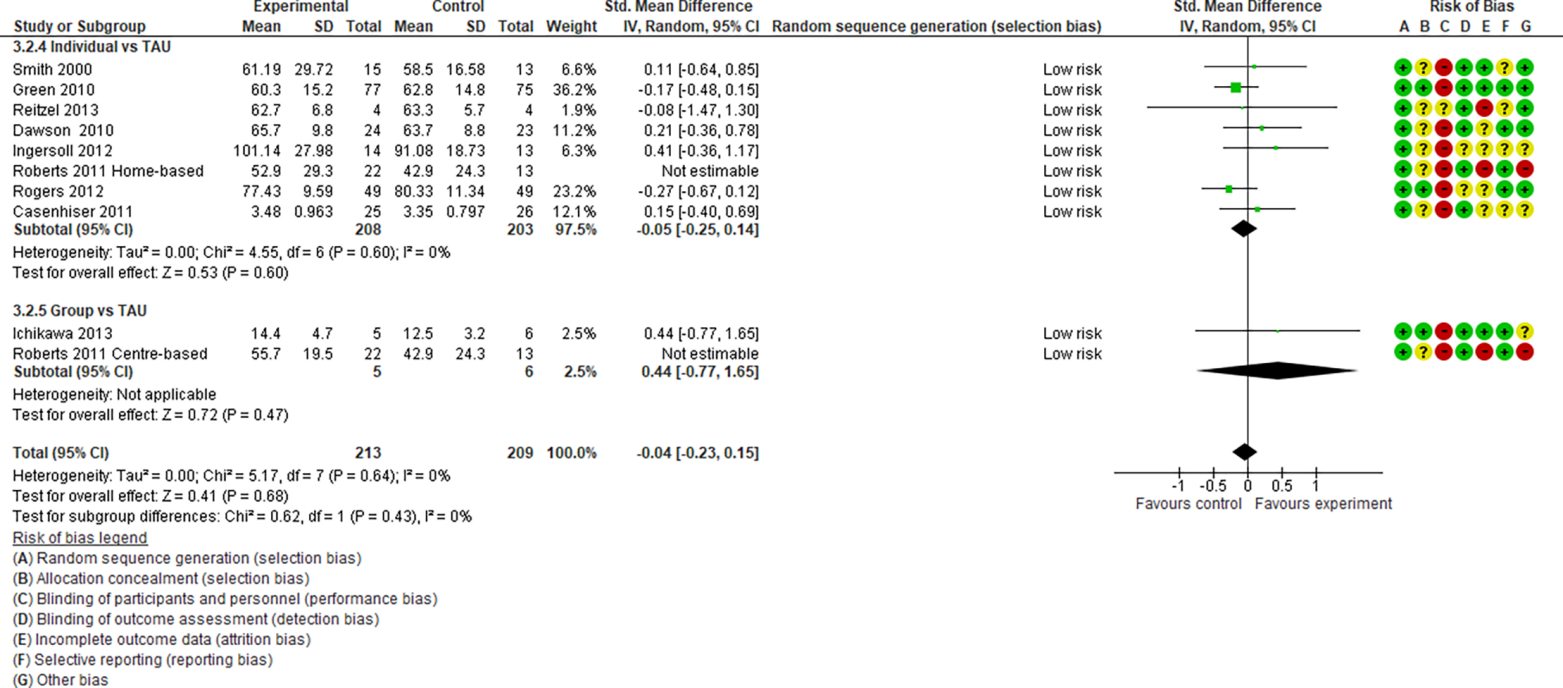
Forest plot of “adaptive behaviour” (Analysis I).

**Fig 10:**
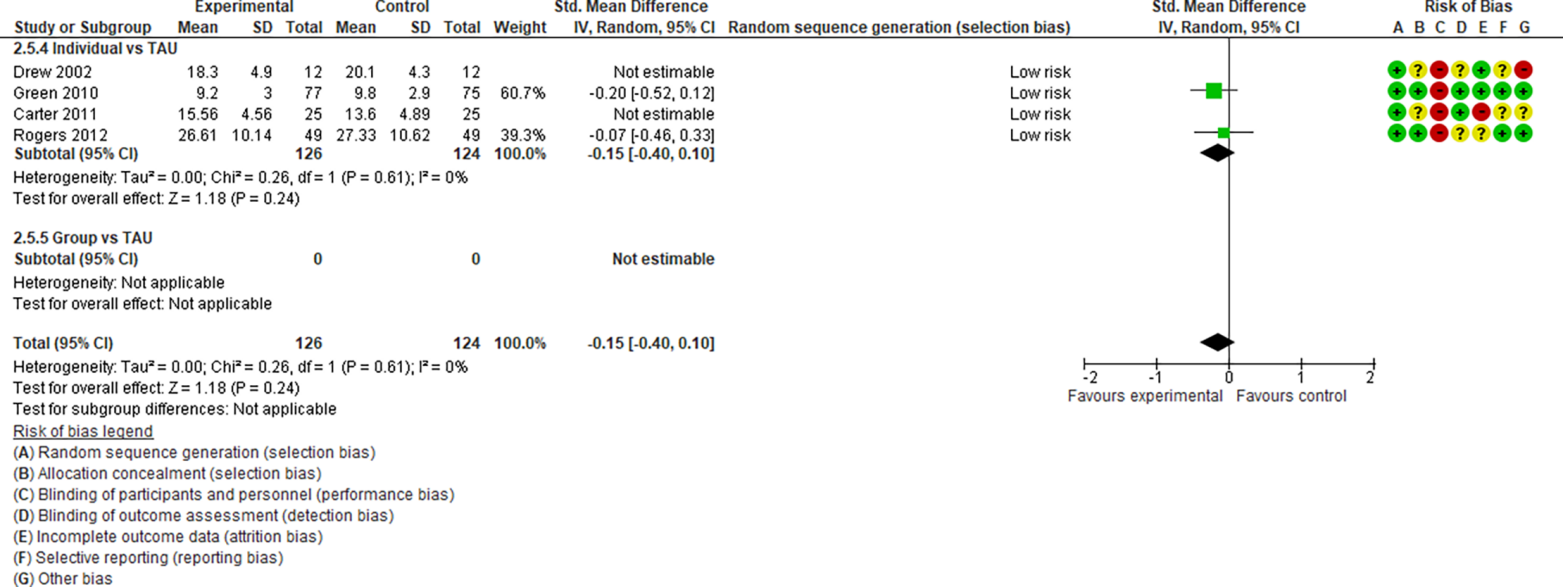
Forest plot of “qualitative impairment in social interaction” (Analysis I).

**Fig 11:**
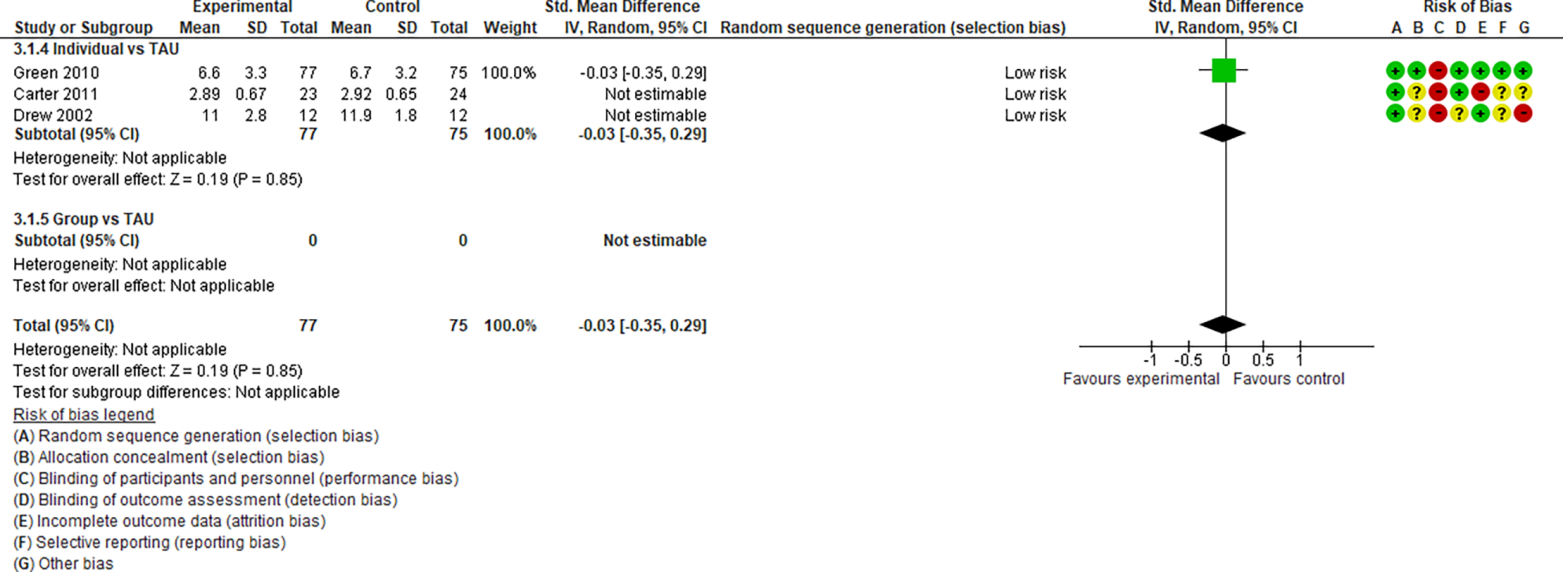
Forest plot of “qualitative impairment in communication” (Analysis I).

**Fig 12:**
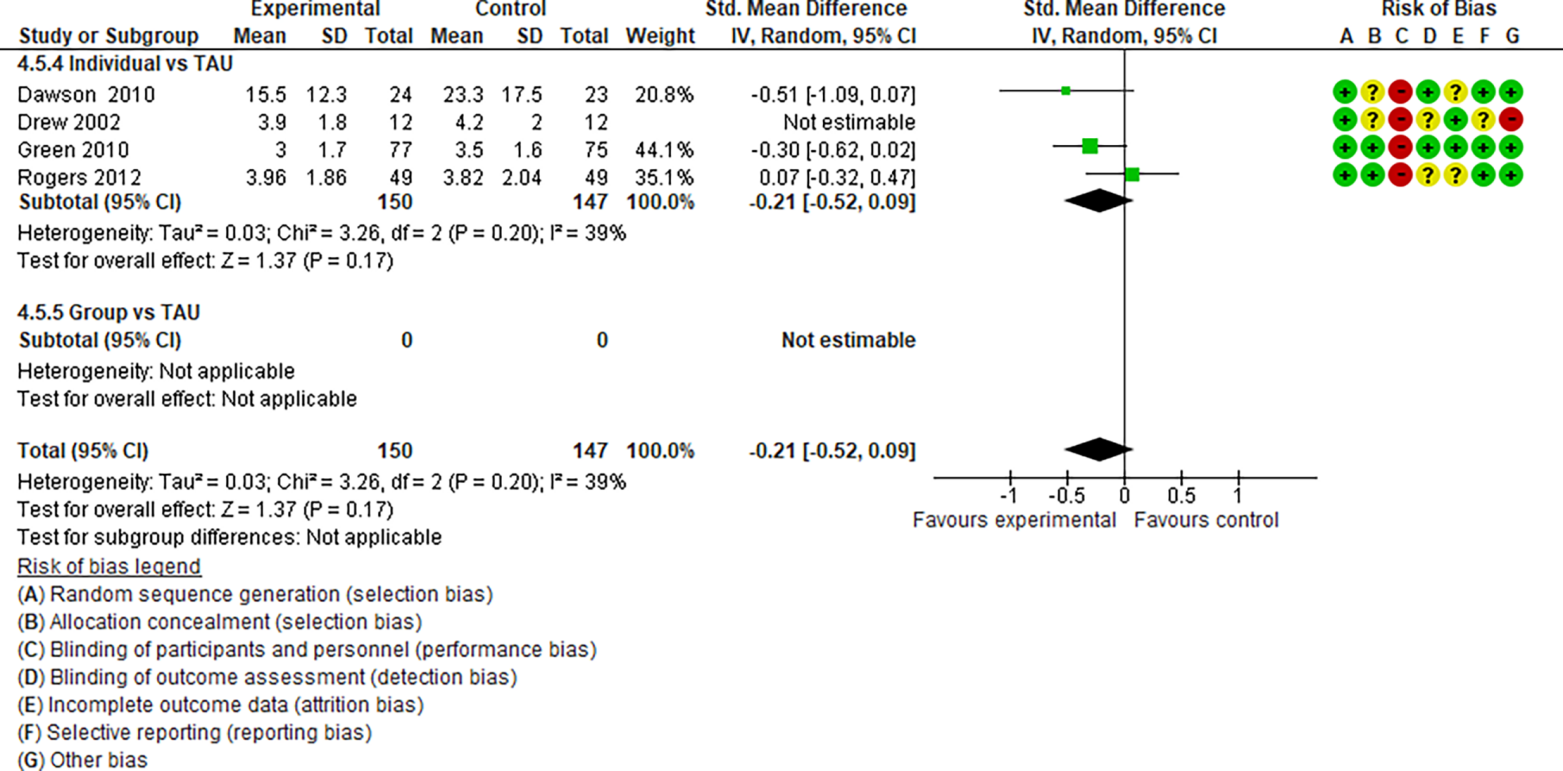
Forest plot of “restricted repetitive and stereotyped patterns of behaviour, interests, and activities” (Analysis I).

**Fig 13:**
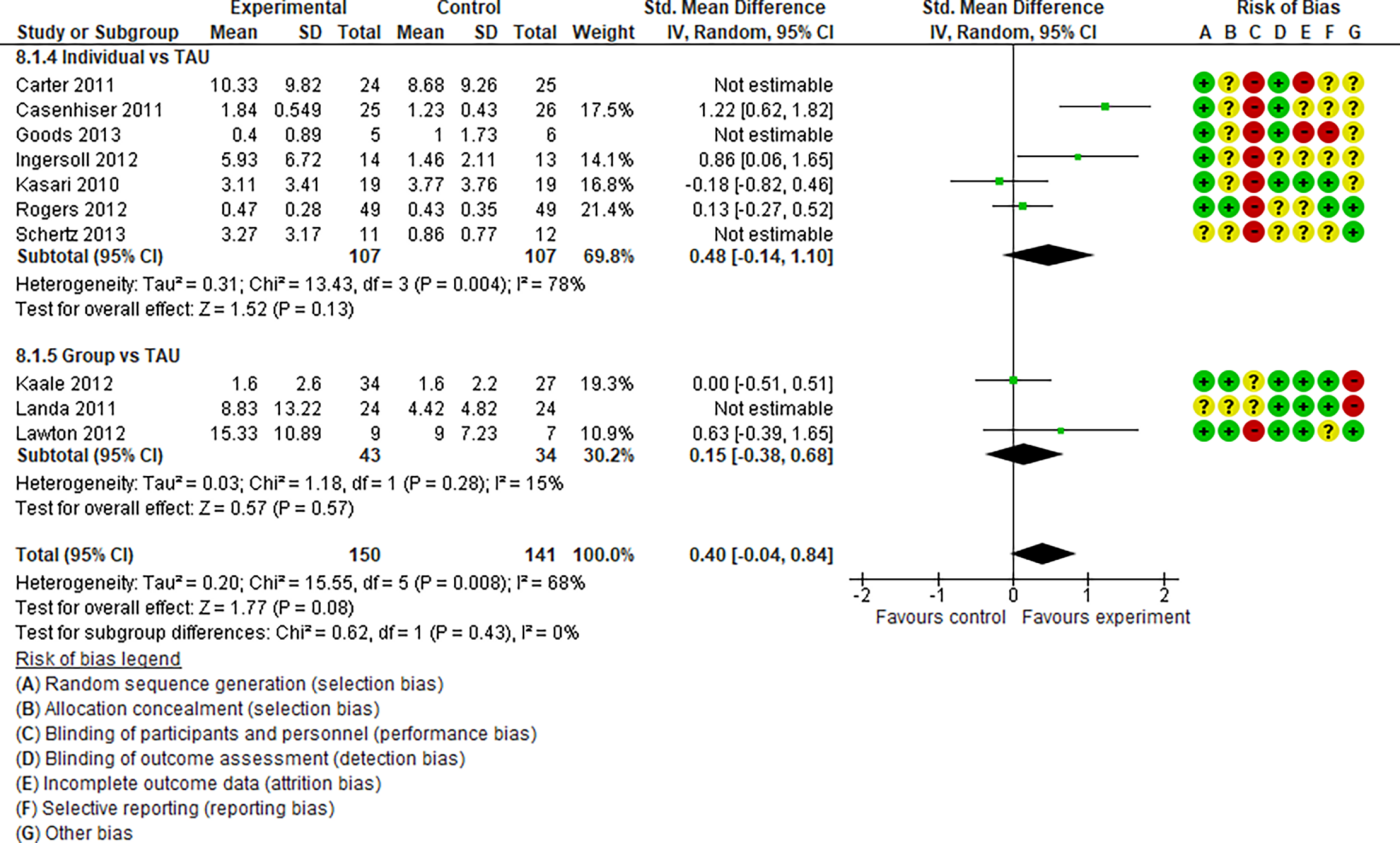
Forest plot of “initiating joint attention” (Analysis I).

**Fig 14:**
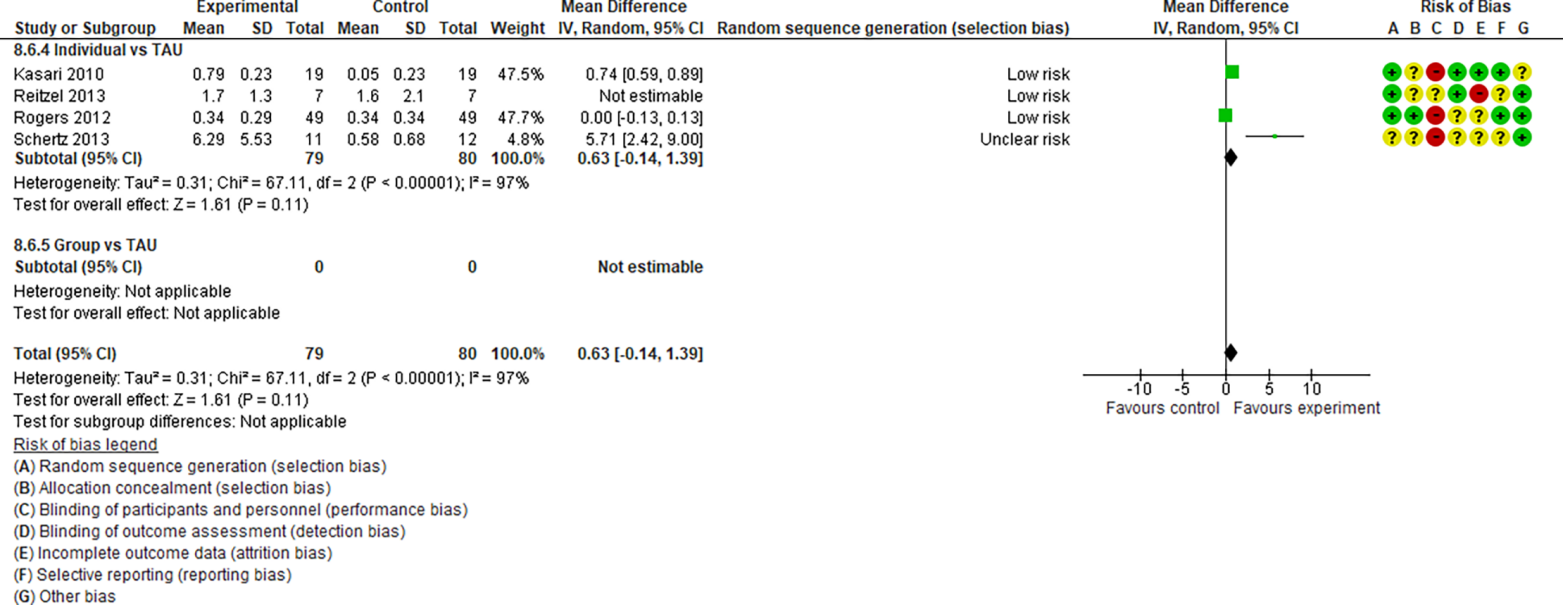
Forest plot of “responding to joint attention” (Analysis I).

**Fig 15:**
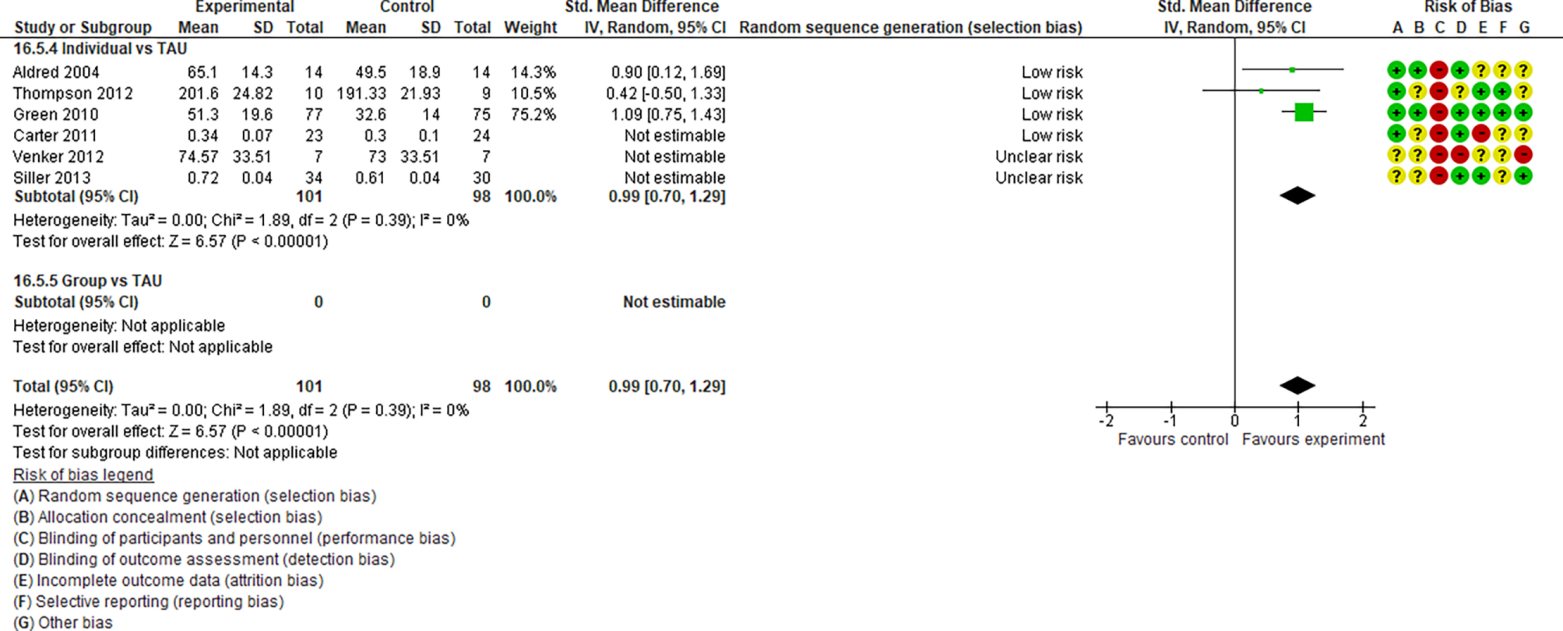
Forest plot of “parental synchrony” (Analysis I).

**Fig 16:**
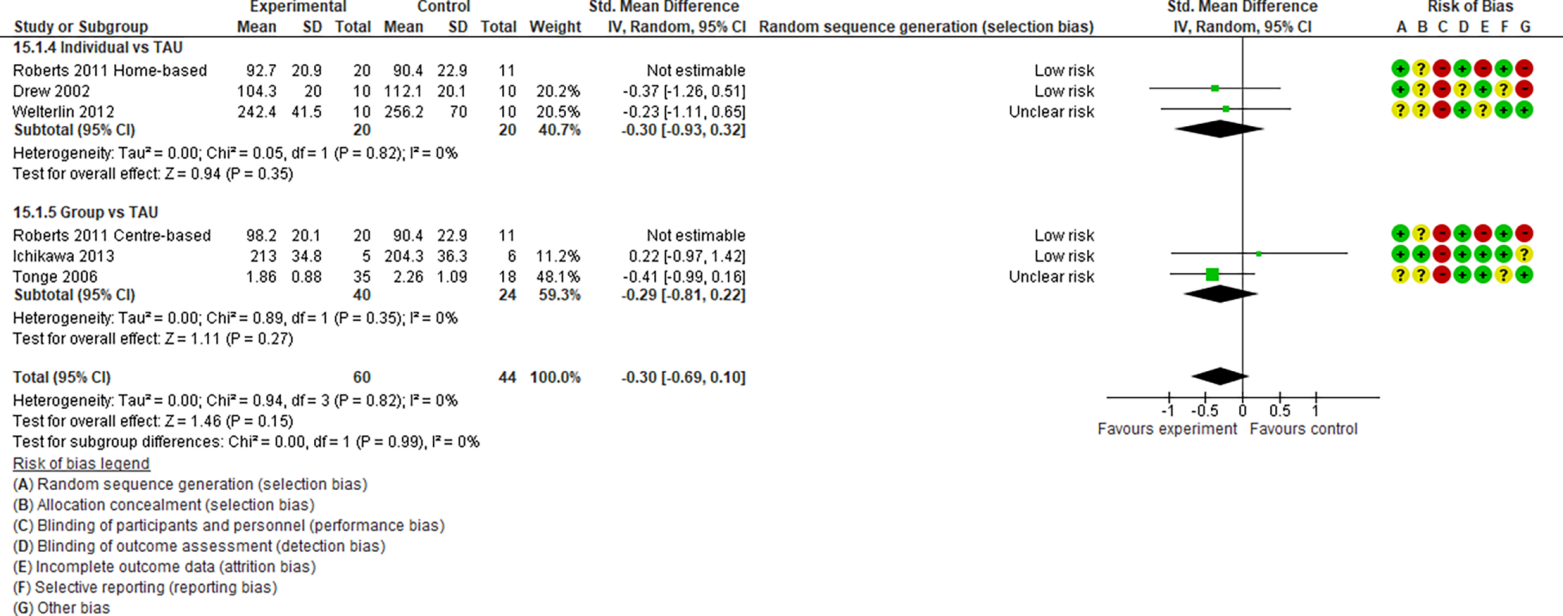
Forest plot of “parenting stress” (Analysis I).

**Fig 17:**
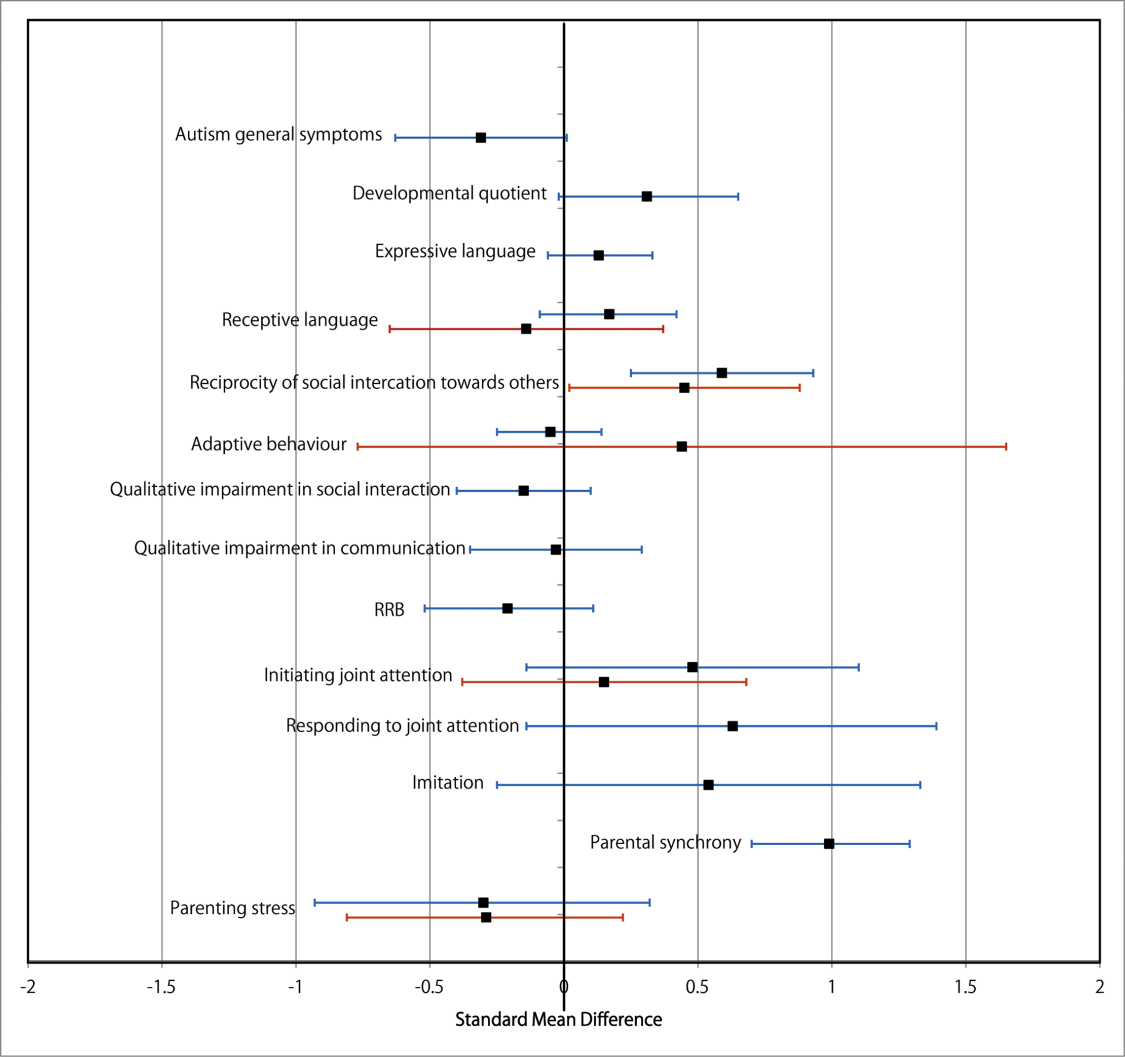
Results of the data synthesis of the effect sizes of the included individual and group intervention studies for each outcome (Analysis I) footnotes: Blue bars indicate the synthesised effect sizes of the individual intervention studies, and red bars indicate the synthesised effect sizes of the group intervention studies. RRB indicates restricted repetitive and stereotyped patterns of behaviour, interests, and activities.

### Participants

Analysis I included a total of 594 children, and Analysis II involved 1220 children. The smallest study in the review had 11 participants [38], while the largest [37] had 152 participants. Children were between 1 and 6 years of age. This study targeted children with ASD who complied with the international typical diagnostic standards. All study participants had a diagnosis of autism or ASD made by the assessing clinician or psychologist based on the DSM-IV-TR, DSM-IV, DSM-III-R, or ICD-10 classification. No studies were included in which children with ASD were diagnosed by DSM-5. Several of the studies used the Autism Diagnostic Interview-Revised (ADI-R), or ADOS, or both to confirm the diagnosis. None of the children had comorbid or debilitating illnesses, such as cerebral palsy, genetic syndromes, diagnosed hearing impairment, diagnosed visual impairment or seizures, or severe psychiatric disorders. Children’s ethnicities were Caucasian, Latino, Caribbean, Hispanic, African, Asian, and other mixed races. The study settings were the USA, Canada, UK, Australia, Japan, Korea, and Thailand.

### Intervention effects

#### Quantitative data synthesis

We conducted meta-analyses for the 16 outcomes examined. Although Tonge et al. 2006 [56] and Tonge et al. 2014 [57] had two intervention conditions (parent education and behaviour management [PEBM] and parent education and counselling [PEC]), the PEC condition was aimed at controlling for nonspecific therapeutic effects compared to the target intervention, PEBM. We therefore did not use the PEC data and included the PEBM data in the data syntheses.

#### Main analysis

Analysis I: Primary outcomes (see Fig 4, Fig 17, Table 2, and S2 Table)**. 1**.1. Autism general symptoms. We rated the overall quality of evidence as “moderate” (see Table 1). Three studies (three individual intervention studies and zero group intervention studies) were included. The overall effect of the individual interventions was not significantly improved compared to the control condition (SMD [95% confidence interval {CI}] = −0.31 [−0.63, 0.01], p = 0.28). We were unable to make comparisons between individual and group interventions with the included studies. There was no significant heterogeneity in the studies included in the analysis (I^2^ = 21%).

**Table 2:** The results of Analysis I for individual and group interventions for each outcome.

		Individual			Group		
	Outcome	p-value	SMD (95%CI)	I^2^(%)	p-value	SMD (95%CI)	I^2^(%)
Primary outcome	Autism general symptoms	0.28	−0.31[−0.63, 0.01]	21	N/A		
Secondary outcomes	Developmental quotient	0.02*	0.36[0.05, 0.66]	20	N/A		
	Developmental quotient (baseline imbalance-adjusted)	0.07	0.31[−0.02, 0.65]	26	N/A		
	Expressive language	0.18	0.13[−0.06, 0.33]	0	0.92	0.11[−0.07, 0.30]	N/A
	Expressive language (baseline imbalance-adjusted)	0.18	0.13[−0.06, 0.33]	0	N/A		
	Receptive language	0.19	0.17[−0.09, 0.42]	28	0.59	−0.14[−0.65, 0.37]	N/A
	Reciprocity of social interaction towards others	p<0.001***	0.59[0.25, 0.93]	18	0.04*	0.45[0.02, 0.88]	18
	Adaptive behaviour	0.60	−0.05[−0.25, 0.14]	39	0.47	0.44[−0.77, 1.65]	N/A
Other outcomes	Qualitative impairment in social interaction	0.61	−0.15[−0.40, 0.10]	0	N/A		
	Qualitative impairment in communication	0.85	−0.03[−0.35, 0.29]	N/A	N/A		
	Restricted repetitive and stereotyped patterns of behaviour, interests and activities	0.17	−0.21[−0.52, 0.09]	39	N/A		
	Initiating joint attention	0.13	0.48[−0.14, 1.10]	78	0.57	0.15[−0.38, 0.68]	15
	Responding to joint attention	0.11	0.63[−0.14, 1.39]	97	N/A		
	Imitation	0.11	0.54[−0.25, 1.33]	62	N/A		
	Parental synchrony	p<0.001***	0.99[0.70, 1.29]	0	N/A		
	Parenting stress	0.35	−0.30[−0.93, 0.32]	0	0.27	−0.29[−0.81, 0.22]	0

“p-value” indicates value of the test of overall synthesis. SMD indicates standard mean difference of the synthesised effect. 95% CI indicates 95% confidence interval of the SMD of the overall synthesis.

* and *** indicate statistically significant effects (p < 0.05, p < 0.01, and p < 0.001, respectively) in the analysis.

N/A indicates data synthesis could not be performed due to lack of available studies.

“baseline imbalance-adjusted” indicates results of the analysis after excluding the studies with baseline imbalances.

Analysis I: Secondary outcomes. 2.1. Developmental quotient (see Fig 5, Fig 17, and Table 2). Five studies (five individual intervention studies and zero group intervention studies) were included [34, 35, 41, 50, 54]. The overall effect of the three individual interventions was significant compared to the control condition (SMD [95% CI)] = 0.36 [0.05, 0.66], p = 0.02). There was no significant heterogeneity in the studies involved in the analysis (I^2^ = 20%). 2.2. Expressive language (see Fig 6, Fig 17, and Table 2). Eight studies (seven individual intervention studies [31, 34, 35, 37, 50, 54, 55] and one group intervention study [43]) were included. The overall effect of the individual intervention studies did not show a significant improvement compared to the control condition (SMD [95% CI] = 0.13 [−0.06, 0.33], p = 0.84). The effect of the group intervention also did not show a significant effect compared to the control condition (SMD [95% CI] = - 0.03[−0.54, 0.48], p = 0.92). There was no significant difference in the effect between the individual interventions and the group intervention (p = 0.56). There was no significant heterogeneity in the intervention effect indicated (I^2^ = 0%). 2.3 Receptive language (see Fig 7, Fig 17, and Table 2). Eight studies (seven individual intervention studies [31, 34, 35, 37, 50, 54, 55] and one group intervention study [43]) were included. The overall effect of the individual intervention studies did not show a significant improvement compared to the control condition (SMD [95% CI] = 0.17 [−0.09, 0.42], p = 0.19). The effect of the group intervention study was also non-significant compared to the control condition (SMD [95% CI] = 0.14 [−65, 0.37], p = 0.59). There was no significant heterogeneity in the intervention effect indicated (I^2^ = 10.0%). 2.4 Reciprocity of social interaction towards others (see Fig 8, Fig 17, and Table 2). Eight studies (five individual intervention studies [31, 33, 37, 55, 66] and three group intervention studies [38, 43, 47]) were included. The overall effects of both the individual and group intervention studies showed significant improvements compared to the control condition (SMD [95% CI] = 0.59 [0.25, 0.93], p = 0.16; 0.45 [0.02, 0.88], p = 0.39, respectively). There was no significant difference in the effect between the individual interventions and the group intervention (p = 0.62). There was no significant heterogeneity in the intervention effect indicated (I^2^ = 18%). 2.5 Adaptive behaviour (see Fig 9, Fig 17, and Table 2). Eight studies (seven individual intervention studies [33, 34, 37, 40, 49, 50, 54] and one group intervention study [38]) were included. The overall effect of neither the individual nor group intervention studies showed significant improvement compared to the control condition (SMD [95% CI] = −0.05 [−0.25, 0.14], p = 0.60; 0.44 [−0.07, 1.65], p = 0.47, respectively). There was no significant difference in the effect between the individual interventions and the group intervention (p = 0.43). There was no significant heterogeneity in the intervention effect indicated (I^2^ = 0%).

See Figs 10–17 and Table 2 for the results of the remaining outcomes (3.1–3.10). There were no significant differences between the individual and group intervention studies for outcomes: 3.1–3.10. “Parental synchrony” showed significant improvement in the overall syntheses of the individual intervention studies [31, 37, 55] compared to the control condition (SMD [95% CI] = 0.99 [0.70, 1.29], p < 0.01).

### Sensitivity analyses

#### Analysis II (see S3 Table and S1 Fig)

The overall effect of the individual intervention showed significant improvement compared to the control condition on “autism general symptoms,” “developmental quotient,” “expressive language,” “reciprocity of social interaction towards others,” and “parental synchrony” (SMD [95% CI] = −0.30 [00.53, −0.08], p < 0.01; 0.23 [0.03, 0.42], p = 0.02; 0.17 [0.01, 0.33], p = 0.04; 0.50 [0.31, 0.69], p < 0.001; and 0.98 [0.30, 1.66], p < 0.01, respectively). There were no outcomes for which group interventions showed significant improvement compared to the control condition. There were no significant differences between the individual and group intervention studies on the outcomes reviewed.

#### Analysis III (see Table 3 and S5 Table)

The overall effect of the intervention condition compared to the control condition for both individual and group interventions showed no significant effects on any of the outcomes.

**Table 3:** The results of sensitivity analyses for Analysis I with cluster-robust variance estimation (Analysis III).

		Data synthesis by random effects model with cluster-robust variance estimation							
		Individual intervention				Group intervention			
	Outcome	Estimate	RSE	95%CI	p-value	Estimate	RSE	95%CI	p-value
Primary outcome	Autism general symptoms	-0.27	0.41	[-5.42,4,88]	0.63	N/A			
Secondary outcomes	Developmental quotient	0.29	0.49	[-6.00,6.57]	0.67	N/A			
	Expressive language	0.14	0.24	[-2.90,3.17]	0.67	N/A			
	Receptive language	0.13	0.40	[-5.02,5.27]	0.81	N/A			
	Reciprocity of social ineteraction towards others	0.52	0.48	[-5.63,6.67]	0.40	0.54	0.39	[-4.48,5.55]	0.48
	Adaptive behavior	-0.05	0.30	[-3.90,3.79]	0.89	N/A			
Other outcomes	Qualitative impairment in social interaction	-0.15	0.16	[-2.21,1.91]	0.53	N/A			
	RRB	-0.21	0.41	[-5.46,5.04]	0.70	N/A			
	Initiating joint attention	0.30	0.44	[-5.35,5.96]	0.74	0.39	0.89	[-10.97,11.75]	0.62
	Responding to joint attention	0.60	2.01	[-24.98,26.17]	0.82	N/A			
	Parental synchrony	0.99	0.35	[-3.40,5.39]	0.21	N/A			
	Parenting stress	-0.11	0.45	[-5.81,5.58]	0.35	-0.30	0.18	[-2.62,2.01]	0.84

Estimates indicate the estimated standard mean difference in the random effects model with cluster-robust variance estimation. RSE indicates robust standard error. CI indicates confidence interval. RRB indicates restricted repetitive and stereotyped patterns of behaviour, interests, and activities. "p-value" indicates p value of the tests of coefficients with cluster-robust variance estimation. N/A indicates data synthesis could not be performed due to lack of available studies.

#### Analysis IV (see Table 4 and S6 Table)

Individual interventions showed significant effects compared to the control condition on “reciprocity of social interaction towards others” and "parental synchrony" (mean estimate [95%CI], robust standard error, p = 0.50 [0.20, 0.81], 0.13, 0.006; and 1.06 [0.08, 2.05], 0.42, 0.04, respectively), and none of the outcomes showed significant effects under the intervention condition compared to the control condition for group interventions.

**Table 4:** A comparison of the effects among Analysis I, II, III and IV on each outcome in terms of the statistical significance.

		Analysis I			Analysis II			Analysis III		Analysis IV	
	Outcome	I	G	I vs G	I	G	I vs G	I	G	I	G
Primary outcome	Autism general symptoms	–	N/A	N/A	**	–	–	–	N/A	–	N/A
Secondary outcomes	Developmental quotient	*	N/A	N/A	*	–	–	–	N/A		N/A
	Developmental quotient (baseline imbalance-adjusted)	–	N/A	N/A	–	–	–	–	N/A	–	N/A
	Expressive language	–	N/A	N/A	*	–	–	–	N/A	–	–
	Expressive language (baseline imbalance-adjusted)	–	N/A	N/A	*	–	–	–	N/A	–	–
	Receptive language	–	–	–	–	–	–	–	N/A	–	–
	Reciprocity of social intercation towards others	***	*	–	***	–	–	–	–	**	N/A
	Adaptive behaviour	–	–	–	–	–	–	–	N/A	–	–
Other outcomes	Qualitative impairment in social interaction	–	N/A	N/A	–	N/A	N/A	–	N/A	–	N/A
	Qualitative impairment in communication	–	N/A	N/A	–	N/A	N/A	–	N/A	–	N/A
	RRB	–	N/A	N/A	–	N/A	N/A	–	N/A	–	N/A
	Initiating joint attention	–	–	–	–	–	–	–	–	–	–
	Responding to joint attention	–	N/A	N/A	–	N/A	N/A	–	N/A	–	N/A
	Parental synchrony	***	N/A	N/A	***	N/A	N/A	–	N/A	*	N/A
	Parenting stress	–	–	–	–	–	–	–	–	–	–

"I" indicates individual interventions. "G" indicates group interventions. "I vs G" indicates the results of statistical comparison of individual vs group interventions. RRB indicates restricted repetitive and stereotyped patterns of behaviour, interests, and activities.

*, **, and *** indicates statistically significant effects (p<0.05, p<0.01, and P<0.001, respectively) in the analysis.

N/A indicates data synthesis could not be performed due to lack of available studies.

"baseline imbalance-adjusted" indicates a sensitivity analysis which excluded a study with a significant baseline imbalance on the outcome.

"–" indicates non-statistical significance.

#### Sensitivity analysis excluding one study with a significant baseline imbalance

For “Developmental quotient”, since Drew et al.’s study [35] had a significant baseline imbalance (mean [SD]: experimental group [Exp.] = 88.1 [11.2], control group [Cont.] = 66.0 [16.5]), we performed sensitivity analyses after removing the study. These analyses showed that the results changed for the statistical significance of the effect on this outcome (subgroup difference: p = 0.87; individual intervention: mean [95% CI] = 0.20 [-0.01, 0.40], p = 0.02; group intervention: 0.25 [-0.32, 0.81], p = 0.40). For “Expressive language”, since there was a significant baseline imbalance in Kaale et al.’s 2012 study [43] (mean [min-max]: experimental group [Exp.] = 14.2 [9.17–19.3], control group [Cont.] = 20.1 [13.5–26.8]) and the home-based programme (mean [SD]: Exp. = 3.4 [8.3], Cont. = 6.0 [10.9]) and the centre-based programme (mean [SD]: Exp. = 8.2 [16.6], Cont. = 6.0 [10.9]) of Roberts et al.’s 2011 study [60], we performed sensitivity analyses after removing those studies. These analyses showed no marked changes in the results regarding the statistical significance of the effect on this outcome (subgroup difference: p = 0.55; individual intervention: mean [95% CI] = 0.19 [0.03, 0.36], p = 0.02; group intervention: 0.08 [-0.27, 0.42], p = 0.66).

## Discussion

### Principal findings

The present study compared the two types of intervention methods (individual vs. group) for pre-school children with ASD. Our meta-analysis of both individual and group interventions showed significant effects on “Reciprocity of social interaction towards others”. The sensitivity analyses including studies that were excluded due to a risk of biases in the main analyses showed significant effects on "autism general symptoms", "expressive language", "reciprocity of social interactions towards others", "parental synchrony" for individual interventions but no significant effects on any outcome for group interventions. The sensitivity analyses of the main analyses by cluster-robust standard errors, which considered multiple dependent variables measured by the included studies, showed no significant effects on any of the outcomes for either individual or group interventions. The sensitivity analyses of all included studies by cluster-robust standard errors, showed significant effects on“reciprocity of social interactions towards others” and “parental synchrony” for individual interventions but no significant effects on any outcomes for group interventions. The results suggested that the two intervention methods did not have significantly different effects on autism general symptoms. The results of the main analyses also suggested that the two intervention types did not have significantly different effects on other secondary outcomes (“developmental quotient,” “expressive language,” “receptive language,” “reciprocity of social interaction towards others,” and “adaptive behaviour”).

### Strengths and weaknesses of the study

To our knowledge, this is the first meta-analysis to compare the effects of individual and group interventions for children with ASD. All of the studies included in the meta-analyses were RCTs, and we applied rigorous exclusion criteria for the data syntheses in Analyses I and III. Thus, the quality of evidence in our study can be regarded as reliable. This conclusion is corroborated by Table 1, in which the quality of evidence for all outcomes was regarded as “moderate” or “high”. We also perform sensitivity analyses rigorously to investigate the rationale for the results of the main analyses.

However, limitations associated with the present study warrant mention. First, the small number of studies, especially for group interventions, limited the ability to compare the effects of individual and group interventions on the outcomes. There was disparity between the total number of individual vs. group intervention studies, as well as the total absence of the group intervention studies that examined many of the outcomes of interest. Second, multiple dependent outcomes of the meta-analyses can cause Type I rate inflation. Type I errors are also common in underpowered meta-analyses [67]. To address this issue, we used random effects model analyses with cluster-robust standard error. Third, since the measurement tools used varied among studies, the data synthesis of some outcomes (“initiating joint attention” and “responding to joint attention”) indicated high heterogeneity. Those results are therefore regarded as unreliable. However, since we used SMD for the data synthesis, which adjusted for measurement diversity, the heterogeneity of the analyses of the remaining outcomes was non-significant, and the results of those outcomes can be regarded as reliable. Fourth, there was a potential confounding factor related to the variability in the intervention approaches that were categorised as either individual or group intervention. Whether one type of intervention was delivered individually or to a group depended on the intervention approach that was selected. For example, some types of intervention are more developmentally-based and/or relationship-based, whereas other types are founded on the principles of applied behaviour analysis. Either of these types of approaches could conceivably be implemented in either individual or group contexts. Such intervention approaches could affect the categorization for either individual or group intervention, which therefore could also affect the findings of meta-analyses. Fifth, the analyses of the present study did not take the intervention dosages into consideration. Relatively long and short dosages of such intervention could thus have over-represented the effects on the outcomes for the data syntheses. This intervention dosage issue could also be a potential confounding factor, and one of the methodological limitations associated with the present study.

### Comparison with other studies

Our finding of a significant effect on the “Reciprocity of social interaction towards others” in Analysis I in both individual and group interventions suggests that this outcome may be a promising effective target for early intervention in children with ASD. However, there were discrepancies in the findings on this outcome between the main analysis and its sensitivity analyses, possibly due to the small number of studies included in the analyses. On the other hand, this outcome can be thought to be a dependent variable. An interventionist might affect the outcome measurement, e.g. the therapist might measure the outcome, or set up, or observe the measurement procedures. In addition, many of the studies included in the present meta-analysis employed programmes with parent-implemented treatments, and the outcomes were thereafter measured in parent-child sessions. When using programmes with parent-implemented treatments and the outcome measurements in parent-child sessions, the parents participating in the intervention condition of those studies would have been taught to intensively elicit the child’s response of the reciprocal exchange, while the parents in the control group would not have received such intensive orientation from the therapists. Since this outcome can function as a dependent variable that might be affected by interventionists and context-bound to interactions with the child's parent, we cannot conclude that the children in the intervention group gained generalised skills for engaging in reciprocal interactions with others, although a significant effect regarding the outcome was observed in meta-analyses. As such, further meta-analyses focusing on parent-mediated intervention studies that measure the effects of “reciprocity of social interaction toward others” via parent-child dyad settings are needed, and whether or not the effects can really be generalised to include both parents and others needs to be confirmed using validated measurements and settings of third-person-child dyads.

Our results also suggest that “parental synchrony” may be a promising target for individual interventions for ASD, which is consistent with our previous study [68]. The data synthesis did not include group intervention studies for this outcome. The involvement of individual intervention studies in this outcome despite no group intervention studies being included may be due to differences in the structure characteristics between the two types of interventions. In individual interventions, therapists usually develop tailor-made programmes with parent-therapist dialogue as well as observation of the child. In the dialogue, therapists will advise parents how they should interact with their child. This advice can help parents enhance their parental synchrony. Therapists may have less opportunity to have a dialogue with parents and offer advice for parent-child interactions in group interventions than in individual interventions. Parental synchrony influences the developmental trajectory of the communication abilities of children with ASD [69]. It will be beneficial for children with ASD and their parents when the therapists of the group interventions encourage parents to increase the degree of parental synchrony.

The results showing significant effects of individual interventions on “autism general symptoms,” “developmental quotient,” “expressive language,” “reciprocity of social interaction towards others,” and “parental synchrony” in Analysis II suggest that individual interventions may be effective for achieving these outcomes. However, these results were inconsistent with those of Analysis III and IV, which considered dependent variables using cluster-robust standard errors. These discrepancies might have been induced by a lack of power due to the small number of studies included in the present meta-analysis. Although intervention studies for children with ASD usually include multiple dependent variables, previous meta-analyses [1, 2, 68, 70–75] did not address this issue. Those results should be re-interpreted from the view of multiple dependent variables.

### Unanswered questions and future research goals

The absence of RCTs regarding group interventions for pre-schoolers with ASD is of practical concern, at least in the many countries that primarily offer community interventions for children with ASD in pre-school group settings (especially for children between 3 to 5 or 6 years of age). Studies that consider both the comparative effectiveness and the cost-effectiveness of group vs. individual ASD interventions can be valuable to people and agencies charged with making decisions on how best to serve young children with ASD from a public health perspective. In addition, to demonstrate the strengths and weaknesses of group compared to individual interventions, further research involving RCTs of group interventions is needed. The present study did not investigate the long-term effectiveness of individual and group interventions for children with ASD. A previous long-term follow-up study of an intervention programme for children with ASD showed significant effectiveness of the programme on improving autism severity [76], although the study did not demonstrate the short-term effectiveness of the programme for this outcome [37]. Whether or not the effects of the present study are effective in the long-term should be investigated by accumulating more follow-up RCTs for meta-analyses. Furthermore, given the increasing volume of individual and group interventions for pre-school children with ASD, periodic updates to the evidence of the present study are also needed.

## Conclusion

The present study suggested the effectiveness of both individual and group interventions in enhancing “reciprocity of social interaction towards others”, and individual intervention on “parental synchrony”. Since the outcome “reciprocity of social interaction towards others” can also be a dependent variable that is usually measured in a context-bound setting with the child's parent, we cannot conclude that individual interventions for pre-school children with ASD have significant effects on their generalised skills at engaging in reciprocal interactions with others, even if those interventions have significant effects on the outcome. However, these outcomes may be promising targets for individual and group interventions involving pre-school children with ASD. The results also suggest that individual intervention may also improve “autism general symptoms” and “expressive language”. The small number of available group intervention studies included in the data synthesis limited our ability to make inferences regarding comparisons of individual versus group interventions for children with ASD.

## Supporting information

S1 Appendix Study protocol. (PDF)

S2 Appendix Search strategy. (PDF)

S3 Appendix Excluded studies. (PDF)

S1 Table Characteristics of the included studies. (PDF)

S2 Table Outcome measure list used in the included studies for the data syntheses. (PDF)

S3 Table The results of Analysis II on each outcome. (PDF)

S4 Table The results of sensitivity analyses for Analysis I with cluster-robust variance estimation (Analysis IV). (PDF)

S5 Table Demographics of the included studies for individual and group interventions for Analysis III. (PDF)

S6 Table Demographics of the included studies for individual and group interventions for Analysis IV. (PDF)

S7 Table PRISMA 2009 checklist. (PDF)

S1 Fig Forest plots of Analysis II. (PDF)
