# Supplementary material for: Meta-analyses of individual versus group interventions for pre-school children with autism spectrum disorder (ASD)
Source: PLoS One. 2018 May 15;13(5):e0196272. doi: 10.1371/journal.pone.0196272 (PMC5953451; doi:10.1371/journal.pone.0196272)
Supplement: S1 Appendix — (PDF) [file pone.0196272.s001.pdf]

S1 Appendix. Study protocol.

Meta-analyses of individual versus group interventions for pre-school children with autism spectrum disorder (ASD): study protocol

Yoshiyuki Tachibana[1, 2, 3], Celine Miyazaki[4], Erika Ota[4, 5], Rintaro Mori[4], Yeonhee Hwang[6], Akiko Terasaka[7], Eriko Kobayashi[8], Yoko Kamio[3]

1. Division of Infant and Toddler Mental Health, Department of Psychosocial Medicine, National Centre for Child Health and Development
2. Smart Aging International Research Center, IDAC, Tohoku University
3. Department of Child and Adolescent Mental Health, National Center for Neurology and Psychiatry
4. Department of Health Policy, National Research Institute for Child Health and Development
5. Department of Global Health Nursing, St. Luke's College of Nursing
6. Department of Education, Tohoku Fukushi University
7. Department of Educational Collaboration, Osaka Kyoiku University
8. TELL Counseling

Correspondence to
Yoshiyuki Tachibana, M.D., Ph.D.,
Division of Infant and Toddler Mental Health, Department of Psychosocial Medicine, National Centre for Child Health and Development
2-10-1 Okura, Setagaya-ku, Tokyo, Japan 157-8535
Tel/Fax: +81 (0) 3 3416 0181; E-mail: tachibana-y@ncchd.go.jp

Ver. 2.4
Date: 07/02/2018

Trial registration:
http://www.crd.york.ac.uk/prospero/register_new_review.asp?RecordID=1349&UserID=230 (CRD42011001349)

Key words
autism spectrum disorder, early intervention, individual, group, meta-analysis, pre-school, child

INTRODUCTION

There has been increasing interest in developing effective interventions for young children with autism spectrum disorder (ASD), since evidence suggests that early intervention programs are indeed beneficial for children with ASD. Intervention programs often improve developmental functioning and decrease maladaptive behaviors and symptom severity [1] and also can improve outcomes in later years for many individuals [2].

An increasing volume of individual intervention programs for pre-school children with ASD has been seen in recent years [3, 4]. On the other hand, group intervention programs are also common [5]. However, there is little rigorous evidence regarding the effects of individual and group interventions on important outcomes. Comparing individual and group interventions and investigating which intervention type will work for specific outcomes will help to identify the approaches that support successful intervention effects for children with ASD. Such an analysis will also reveal how evidence-based assessments of effective methods can be introduced as treatments for children with ASD. Findings of this analysis will aid families, clinicians, and policymakers in determining the appropriate choice of interventions for children with ASD. The results will also help intervention developers to understand the weak points of their interventions and add supplemental approaches to improve those weaknesses.

In this study, we will categorize previously conducted randomized controlled trials (RCTs) of intervention programs for children with ASD into two groups: individual and group interventions. We hypothesize that these two intervention types will have significant differences on certain outcomes. The objective of our study is to test this hypothesis and conduct a meta-analysis of methodologically adequate studies in accordance with the Cochrane Collaboration’s Systematic Reviews [6], which allows, for the first time, a comparison of group versus individual interventions on important

outcome measures. We also investigate the effects of individual versus group interventions on important outcomes.

METHODS

This study will be performed as a sub-analysis of our previous study [7]. The methods used to conduct this study will be in accordance with the Cochrane Handbook for Systematic Reviews [6]. The PRISMA guidelines [8, 9] will be used to prepare this review.

Selection criteria

Types of studies

We will include RCTs, quasi-RCTs, and crossover trials.

Types of participants

Participants will be children aged 6 years old or younger, with a diagnosis of ASD as below.

Diagnostic and Statistical Manual of Mental Disorders Third Edition - Revised (DSM-III-R) [10]

Diagnostic and Statistical Manual of Mental Disorders Fourth Edition (DSM-IV) [11]

Diagnostic and Statistical Manual of Mental Disorders-IV-Text Revision (DSM-IV-TR) [12]

- Autistic disorder
- Asperger disorder
- Pervasive developmental disorder not otherwise specified (PDD-NOS)

International Classification of Diseases-10 (ICD-10) [13]

S1 Appendix. Study protocol.

- Childhood autism
- Asperger syndrome, atypical autism
- Other pervasive developmental disorders
- Pervasive developmental disorders, unspecified.

*Diagnostic and Statistical Manual of Mental Disorders-5 (*DSM-5*)*[14].

- Autism spectrum disorder

Types of interventions

Two types of interventions will be targeted in this study: individual and group interventions. Individual interventions are defined as those which consisted of individual sessions in which the therapist intervenes one-on-one. Group interventions are defined as those which consisted of group sessions without individual sessions. Studies with interventions delivered to the parents/guardians and/or directly to the child by special educators, teachers, speech pathologists, psychologists, or other allied health professional students will be included. We will classify the studies reviewed into these two types of interventions and limited the analysis to pre-school children aged 6 years or younger.

Studies describing pharmacological, alternative, or complementary medicine interventions will be excluded. Studies in which the control group received a specific early intervention for children with ASD, which are considered "treatment as usual" provided by their local services, will be excluded.

Types of outcomes

A feature of this review is that we will systematically classify the various outcome measures used within recent intervention trials. Among many outcomes, we

prioritize autism symptoms as the primary outcome (see I below). In addition, we will investigate other relevant secondary outcomes (see II below), which are important for children's daily lives and prognoses. We choose outcomes that had been investigated in previous studies [15-21].

I. Primary outcomes

1.1. Autism general symptoms

This outcome indicates the severity of autism symptoms related to the definitional symptoms of autistic disorder in DSM-IV-TR; the comparison score of the Autism Diagnostic Observation Schedule (ADOS) [22, 23] will be used for these outcomes.

II. Secondary outcomes

Nonspecific developmental outcomes, which are not required for the definition of autism diagnosis but are used in some studies, will be used for the data syntheses. Here we define "developmental quotient" as a combination of developmental quotient and intelligence quotient. In this paper, we will report five outcomes, as below. "Reciprocity of social interaction towards others" is defined as a child's reciprocal social interactions with his/her parents or examiners.

2.1. Developmental quotient

2.2. Expressive language

2.3. Receptive language

2.4. Reciprocity of social interaction towards others

2.5. Adaptive behavior

In addition, we will also report other outcomes as follows. Parental synchrony (3.9) is defined as parental synchronous response to the child.

S1 Appendix. Study protocol.

3.1. Qualitative impairment in social interaction

3.2. Qualitative impairment in communication

3.3. Restricted repetitive and stereotyped patterns of behavior, interests, and activities

3.4. Initiating joint attention

3.5. Responding to joint attention

3.6. Parental synchrony

3.7. Parenting stress

Regarding autism symptom outcomes 1.1, 3.1, 3.2, and 3.3, studies without objective coding of child observation (e.g., only using parent questionnaire or parent interview) will be excluded from the analyses.

Electronic search

We searched the following databases: PsycINFO, Medline via Ovid, ERIC, CINHAL, and the Cochrane Central Register of Controlled Trials (CENTRAL) without any language restriction on October 2, 2014.

We used the following search terms to search all trial registries and databases: “autism,” “autism spectrum disorder,” “ASD,” “high function autism,” “high function ASD,” “Asperger syndrome,” “pervasive developmental disorder,” “PDDNOS,” “intervention,” “treatment,” “therapy,” “communication,” “interpersonal,” “speech,” “interaction,” “synchrony,” “relationship,” “language,” “social,” “development,” “behavior,” “intensive behavioral intervention,” “trial,” and “outcome.” The search was limited by child age, including only those aged 0–6 years, and by study type (“randomized controlled trial”). This search strategy was peer-reviewed by the librarians of the University of Manchester and the National Research Centre for Child Health and Development. Other relevant studies were also searched for from reference lists to

identify trials and review articles. ClinicalTrials.gov and the Cochrane Library websites were also searched for randomized trials that were registered as completed but not yet published.

Search of other resources

Reference lists from identified trials and review articles were manually scanned to identify any other relevant studies. ClinicalTrials.gov and CENTRAL were also searched for RCTs that were registered as completed but not yet published.

Data collection and analysis

All references found by the search strategy were gathered by the reference-management program EndNote X6. Two authors (AT and EK) independently reviewed abstracts of potentially relevant studies. All citations sourced from the search strategy were transferred to EndNoteX6 (Thomson Reuters, New York City, NY, USA), a reference-management database software. Initial screening of titles and abstracts by EK eliminated all citations that were obviously irrelevant to the topic (e.g., prevalence studies, studies not relating to autism spectrum disorders, and single case studies). Thereafter, two of five review authors (YT, YH, EK, CM, and AT) assessed and selected studies for inclusion from the group of potentially relevant studies. In the event of a disagreement, resolutions were reached through discussion with a third referee (EK or YT), if necessary, following inspection of the full paper.

Data extraction and management

S1 Appendix. Study protocol.

EK and CM independently extracted data from selected trials using a specially designed data-extraction form. Extracted data consisted of methods (dose and frequency of intervention); diagnostic description of participants; and type of intervention including target, intensity, duration, and method of application (parent-mediated, therapist, school-based, etc.). Data were extracted independently by two review authors (EK and CM), and disagreements were resolved by negotiation with a third author (YT).

Assessment of risk of bias

Risk of bias was assessed by five independent review authors (YT, YH, EK, AT, and CM), and disagreements were resolved by negotiation with a third review author (YT or EK). We used the Cochrane Collaboration's tool for assessing risk of bias for each included study [24]. The tool included the following domains: sequence generation, allocation concealment, blinding of participants and personnel, blinding of outcome assessment, complete outcome data, selective outcome reporting, and other sources of bias. The process involved recording the appropriate information for each study (e.g., detailed description of the method used to conceal allocation) and evaluating whether there was risk of bias in that domain (e.g., whether allocation was adequately concealed). We judged potential risk of bias for each domain of each study: "low risk of bias," "unclear risk of bias," and "high risk of bias." Whether a study should be included in the meta-analyses was judged individually based on the results of the risk of bias assessment, and studies judged to be at a high risk of bias were excluded.

Management of missing data

For each study, missing data will be handled as follows: when there will be a significant quantity of participant data missing in the report, such that we agree that the

conclusions of the study will be compromised, the trial authors will be contacted. If a reply was not forthcoming or full data will not be made available, those studies will be excluded from the final analysis. We will report reasons for missing data provided by the included studies. The extent to which the results of the review can be altered by the missing data were assessed and discussed. If summary data were missing, trial authors will be contacted.

Data synthesis

Data syntheses will be performed using Review Manager version 5.3 (Cochrane Collaboration software). We will assess continuous data, which will be analyzed on the basis that means and standard deviations (SDs) will be available and that there will be no clear evidence of skew in the distribution. Assuming that two or more studies that will be suitable for inclusion will be found and that the studies will be considered to be satisfactory, a meta-analysis will be performed on the results. To compare the two types of interventions (i.e., group versus individual), we will categorize the studies into subgroups based on the two intervention models described above. Since the reviewed studies measured several outcomes in a nonuniform manner, outcome data will be synthesized using standardized mean difference (SMD; mean divided by SD post-intervention) for both intervention and control groups. Syntheses will be based on previous meta-analyses of interventions for children with ASD [3, 4]. We will synthesize the various categories of outcome measures using an SMD for both groups. We will test the two subgroup interactions (i.e., individual versus group interventions) using the inverse variance method in a random-effects model [24]. We will use the I^2 statistic to assess the rationale of data synthesis based on the degree to which there are heterogeneity in the types of measurement in the included studies [24].

We will also perform an overall synthesis of the included studies for the two intervention types on each outcome. We will analyze these studies in the same way in which previous meta-analyses amalgamated studies of children with ASD, which had different intervention modalities and different measurements [3, 4].

Main Analysis

Analysis I (analysis excluding studies at high risk of bias using a random-effects model)

We will perform data syntheses, excluding studies that were assessed to have a “high” or “unclear” risk of bias in both “random sequence generation (selection bias)” or “allocation concealment (selection bias)” and studies with a “high” risk of bias in “incomplete outcome data (attrition bias).” We will also perform sensitivity analyses, as described below. To interpret the results of the present study, we will prioritize Analysis I. If there was a discrepancy in the results of Analysis I and the sensitivity analyses, we carefully will assess the reasons for these discrepancies in our interpretation.

Sensitivity Analysis

Analysis II (analysis of all included studies)

We will undertake a sensitivity analysis with all the included studies to explore the extent to which studies excluded in Analysis I can affect results of the meta-analysis.

S1 Appendix. Study protocol.

Sensitivity analysis excluding studies with a significant baseline imbalance

If a study included in a meta-analysis has a significant baseline imbalance in a measured outcome between the intervention and control groups, we will also perform a sensitivity analysis with that study excluded.

“Summary of findings for main outcomes” table

We will create a “Summary of findings for main outcomes” table, which shows the main outcomes of this review which may be important to parents, clinicians, and decision makers. We will use the Grades of Recommendation, Assessment, Development and Evaluation (GRADE) system [25] to describe the quality of evidence and the strength of recommendation and used GRADEpro software [26] to construct the tables. We will express the quality of evidence on a four-point adjectival scale (“high,” “moderate,” “low,” or “very low”). EO will code the GRADE scales for the main outcomes of this study.

Authors’ contribution

YT originated the protocol. YT and EO developed a search strategy. YT also drafted this manuscript with the support of CM, EO, RM, and YK. YT, HY, EK, CM, and AT conducted quality assessments. EK, YT, and CM conducted data extraction. The database of individual study data will be developed by EK, YT, and CM. The analyses will be conducted by YT with the support of EK, CM, EO, and RM. All authors will read and approved the final manuscript. EO will code GRADE using the results of this study.

S1 Appendix. Study protocol.

Competing Interests

The researchers of the present study declare that no competing interests exist.

Funding

This study will be supported by the Health and Labour Science Research Grant on Children, Youth and Families (H28-Sukoyaka-Ippan-005 and H29-Sukoyaka-Ippan-004) and the Health and Labour Science Research Grant on Psychiatry and Neurological Disease and Mental Health (26350301) provided by Japan’s Ministry of Health, Labour and Welfare.

Reference

1. Rogers, S.J. and L.A. Vismara, *Evidence-based comprehensive treatments for early autism.* Journal of clinical child and adolescent psychology: the official journal for the Society of Clinical Child and Adolescent Psychology, American Psychological Association, Division 53, 2008. **37**(1): p. 8.
2. United States Government Accountability Office, *Special education: children with autism; report to the Chairman and Ranking Minority Member, Subcommittee on Human Rights and Wellness, Committee on Government Reform, House of Representatives*. 2005, Darby, PA: DIANE Publishing.
3. Fletcher‐Watson, S., et al., *Interventions based on the Theory of Mind cognitive model for autism spectrum disorder (ASD).* The Cochrane Library, 2014.
4. Oono, I.P., E.J. Honey, and H. McConachie, *Parent‐mediated early intervention for young children with autism spectrum disorders (ASD).* Evidence‐Based Child Health: A Cochrane Review Journal, 2013. **8**(6): p. 2380-2479.
5. National Collaborating Centre for Mental, H., *National Institute for Health and*

Care Excellence: Guidance, in *Autism: The Management and Support of Children and Young People on the Autism Spectrum*. 2013, British Psychological Society. Copyright (c) National Collaborating Centre for Mental Health, 2013.: Leicester (UK).

6. Higgins, J.P. and S. Green, *Cochrane handbook for systematic reviews of interventions*. Vol. 5. 2008: Wiley Online Library.

7. Tachibana, Y., et al., *A systematic review with meta-analysis of comprehensive interventions for preschool children with autism spectrum disorder (ASD): study protocol*. BMJ open, 2012. **2**(2): p. e000679: http://bmjopen.bmj.com/content/2/2/e000679.full.

8. Moher, D., et al., *Preferred reporting items for systematic reviews and meta-analyses: the PRISMA statement*. Annals of internal medicine, 2009. **151**(4): p. 264-269.

9. Liberati, A., et al., *The PRISMA statement for reporting systematic reviews and meta-analyses of studies that evaluate health care interventions: explanation and elaboration*. PLoS medicine, 2009. **6**(7): p. e1000100.

10. Association, A.P., *Diagnostic and Statistical Manual of Mental Health Disorders (DSM-III-R)*. 1987: American Psychiatric Association.

11. Edition, F. and A.P. Association, *Diagnostic and statistical manual of mental disorders*. 1994: Washington, American Psychological Association.

12. American Psychiatric Association, *Diagnostic and statistical manual of mental disorders: DSM-IV-TR*. 2000, Arlington, VA: American Psychiatric Publishing, Inc.

13. World Health Organization, *The ICD-10 classification of mental and behavioural disorders: diagnostic criteria for research*. 1993, Geneva: World Health Organization.

14. Association, A.P., *Diagnostic and statistical manual of mental disorders, (DSM-5®)*. 2013: American Psychiatric Pub.
15. Lord, C., et al., *The Autism Diagnostic Observation Schedule—Generic: A standard measure of social and communication deficits associated with the spectrum of autism.* Journal of autism and developmental disorders, 2000. **30**(3): p. 205-223.
16. Turygin, N., et al., *The effect of DSM-5 criteria on the developmental quotient in toddlers diagnosed with autism spectrum disorder.* Developmental Neurorehabilitation, 2013. **16**(1): p. 38-43.
17. Lord, C., S. Risi, and A. Pickles, *Trajectory of Language Development in Autistic Spectrum Disorders.* 2004.
18. Mundy, P., M. Sigman, and C. Kasari, *A longitudinal study of joint attention and language development in autistic children.* Journal of autism and developmental disorders, 1990. **20**(1): p. 115-128.
19. Hastings, R.P. and E. Johnson, *Stress in UK families conducting intensive home-based behavioral intervention for their young child with autism.* Journal of autism and developmental disorders, 2001. **31**(3): p. 327-336.
20. Davis, N.O. and A.S. Carter, *Parenting stress in mothers and fathers of toddlers with autism spectrum disorders: Associations with child characteristics.* Journal of autism and developmental disorders, 2008. **38**(7): p. 1278-1291.
21. Rogers, S.J., *Interventions that facilitate socialization in children with autism.* Journal of autism and developmental disorders, 2000. **30**(5): p. 399-409.
22. Lord, C., et al., *The Autism Diagnostic Observation Schedule—Generic: A standard measure of social and communication deficits associated with the spectrum of autism.* Journal of autism and developmental Disorders, 2000. **30**(3): p. 205-223.

23. Lord, C., et al., *Autism diagnostic observation schedule: ADOS manual*. 2008: Western Psychological Services Los Angeles.

24. Higgins, J. and S. Green, *Cochrane Handbook for Systematic Reviews of Interventions Version 5.1. 0 [updated March 2011]*. 2011, Oxford: The Cochrane Collaboration.

25. Guyatt, G.H., et al., *GRADE: an emerging consensus on rating quality of evidence and strength of recommendations.* Bmj, 2008. **336**(7650): p. 924-926.

26. GRADEpro2014, *McMaster University. GRADEpro.. 3.6. McMaster University, 2014.* 2014.

Changes to the protocol

Methods

Analysis III and IV (analysis which used robust standard error)

We also performed sensitivity analyses of the overall effects on the outcomes for Analysis I and II (Analysis III and IV, respectively). This was because many of the studies had multiple dependent variables that were analysed and were nonindependent of effect sizes. This affected the confidence interval around the summary effect sizes, which could result in Type I error rate inflation. To address this issue, we analysed the data using sensitivity analyses by fitting random effects models with robust standard errors by clusters of internally correlated effect estimates using the SAS software programme, version 9.4 (SAS Institute Inc., Cary, NC, USA).
