# Supplementary material for: Meta-analyses of individual versus group interventions for pre-school children with autism spectrum disorder (ASD)
Source: PLoS One. 2018 May 15;13(5):e0196272. doi: 10.1371/journal.pone.0196272 (PMC5953451; doi:10.1371/journal.pone.0196272)
Supplement: S2 Appendix — (PDF) [file pone.0196272.s002.pdf]

S2 Appendix. Search strategy.

MEDLINE

1 exp Child Development Disorders, Pervasive/
2 Autistic.tw.
3 autism.tw.
4 ASD.tw.
5 Asperger*.tw.
6 pervasive developmental disorder.tw.
7 PDDNOS.tw.
8 PDD--NOS.tw.
9 or/1--8
10 intervention*.tw.
11 treat*.tw.
12 therap*.tw.
13 exp Communication/
14 communication*.tw.
15 interpersonal*.tw.
16 Speech/
17 Speech*.tw.
18 interact*.tw.
19 synchron*.tw.
20 relationship*.tw.
21 language*.tw.
22 social.tw.
23 behaviour*.tw.
24 behavior*.tw.
25 Behavior Therapy/
26 Behavior/
27 ABA.tw.
28 development.tw.
29 modification.tw.
30 or/10--29
31 9 and 30
32 Child, Preschool/
33 child*.tw.
34 preschool*.tw.
35 kindergarten*.tw.
36 Nurseries/
37 Schools,nursery/
38 nurser*.tw.
39 Infant/
40 infan*.tw.
41 baby.tw.
42 babies.tw.
43 toddler*.tw.
44 or/32--43

45	31 and 44
46	randomized controlled trial.pt.
47	controlled clinical trial.pt.
48	randomized.ab.
49	placebo.ab.
50	randomly.ab.
51	trial.ab.
52	groups.ab.
53	or/46--52
54	45 and 53
55	exp Animals/ not Humans.sh.
56	54 not 55
57	remove duplicates from 56
58	limit 57 to yr=""1860 -- Current""

EMBASE
#1
#1.1 'autism'/exp
#1.2 'autism':ab OR 'autism':ti
"#1.3 'asd':ab,ti"
"#1.4 'pervasive developmental disorders':ab,ti"
"#1.5 'autistic':ab,ti"
"#1.6 asperger*:ab,ti"
"#1.7 'pddnos':ab,ti"
"#1.8 'pdd nos':ab,ti"
#1.9 #1.1 OR #1.2 OR #1.3 OR #1.4 OR #1.5 OR #1.6 OR #1.7 OR #1.8
"#1.10 intervention*:ab,ti"
"#1.11 treat*:ab,ti"
"#1.12 therap*:ab,ti"
"#1.13 communication*:ab,ti"
"#1.14 'interpersonal':ab,ti"
#1.15 'speech'/exp
"#1.16 speech*:ab,ti"
"#1.17 interact*:ab,ti"
"#1.18 synchron*:ab,ti"
"#1.19 relationship*:ab,ti"
#1.20 'language'/de
#1.21 'language development'/de
#1.22 'language processing'/exp
"#1.23 language*:ab,ti"
"#1.24 social:ab,ti"
#1.25 'behavior'/exp
"#1.26 behaviour*:ab,ti"
"#1.27 behavior*:ab,ti"
#1.28 'therapy'/exp
"#1.29 'aba':ab,ti"
#1.30 'development'/de
#1.31 'behavior therapy'/exp
#1.32 'behavior modification'/de
"#1.33 modification*:ab,ti"
"#1.34 development*:ab,ti"
#1.35 #1.10 OR #1.11 OR #1.12 OR #1.13 OR #1.14 OR #1.15 OR #1.16 OR
#1.17 OR #1.18 OR
#1.19 OR #1.20 OR #1.21 OR #1.22 OR #1.23 OR #1.24 OR #1.25 OR #1.26 OR #1.27
OR
#1.28 OR #1.29 OR #1.30 OR #1.31 OR #1.32 OR #1.33 OR #1.34
#1.36 #1.9 AND #1.35
#1.37 'child'/exp
#1.38 'nursery'/de
#1.39 'nursery school'/exp
#1.40 'kindergarten'/de

"#1.41 'preschool':ab,ti"
"#1.42 infan*:ab,ti"
"#1.43 baby:ab,ti"
"#1.44 babies:ab,ti"
"#1.45 kindergarten*:ab,ti"
"#1.46 child*:ab,ti"
"#1.47 nurser*:ab,ti"
"#1.48 toddler*:ab,ti"
#1.50 #1.36 AND #1.49
#1.49 #1.37 OR #1.38 OR #1.39 OR #1.40 OR #1.41 OR #1.42 OR #1.43 OR #1.44 OR #1.45 OR
#1.46 OR #1.47 OR #1.48
#1.51 random*:ti OR random*:ab OR factorial*:ti OR factorial*:ab OR 'cross over':ti OR 'cross over':ab OR crossover*:ti OR crossover*:ab OR placebo*:ti OR placebo*:ab OR (doubl*:ti AND blind*:ti) OR (doubl*:ab AND blind*:ab) OR (singl*:ti AND blind*:ti) OR (singl*:ab AND blind*:ab) OR assign*:ti OR assign*:ab OR volunteer*:ti OR volunteer*:ab OR
'crossover procedure'/exp OR 'crossover procedure' OR 'double-blind procedure'/exp OR
'double-blind procedure' OR 'single-blind procedure'/exp OR 'single-blind procedure' OR 'randomized controlled trial'/exp OR 'randomized controlled trial' OR allocat*:ti OR allocat*:ab
#1.52 #1.50 AND #1.51
#1.53 #1.52 NOT ([animals]/lim NOT [humans]/lim)
#1.54 #1.53 AND [embase]/lim NOT [medline]/lim
#2 #1 AND [1-1-1966]/sd NOT [27-9-2014]/sd

PsychoINFO

1	Autism/
2	Pervasive Developmental Disorders/
3	ASD.tw.
4	autism.tw.
5	autistic.tw.
6	Aspergers syndrome/
7	asperger*.tw.
8	PDDNOS.tw.
9	PDD-‑NOS.tw.
10	or/1-‑9
11	exp Intervention/
12	exp Treatment/
13	treat*.tw.
14	therap*.tw.
15	exp Communication/
16	communication*.tw.
17	interpersonal*.tw.
18	speech*.tw.
19	interact*.tw.
20	relationship*.tw.
21	Language/
22	language*.tw.
23	social.tw.
24	behaviour*.tw.
25	behavior*.tw.
26	Behavior Analysis/
27	behavior Modification/
28	Behavior Therapy/
29	modification*.tw.
30	ABA.tw.
31	or/11-‑30
32	10 and 31
33	child*.tw.
34	exp Preschool students/
35	preschool*.tw.
36	infan*.tw.
37	baby.tw.
38	babies.tw.
39	toddler*.tw.
40	kindergarten*.tw.
41	Kindergarten students/
42	Kindergartens/
43	Nursery schools/
44	nurser*.tw.
45	or/33-‑44

46	32 and 45
47	Treatment Effectiveness Evaluation/
48	exp Treatment Outcomes/
49	Psychotherapeutic outcomes/
50	Placebo/
51	exp Followup Studies/
52	placebo$.tw.
53	random$.tw.
54	comparative stud$.tw.
55	randomi#ed controlled trial$.tw.
56	(clinical adj3 trial$).tw.
57	(research adj3 design).tw.
58	(evaluat$ adj3 stud$).tw.
59	(prospectiv$ adj3 stud$).tw.
60	((singl$ or doubl$ or trebl$ or tripl$) adj3 (blind$ or mask$)).tw.
61	control$.tw.
62	or/47--61
63	46 and 62
64	exp Animals/ not Humans.sh.
65	63 not 64
66	remove duplicates from 65
67	limit 66 to yr="1860 --Current"
68	limit 66 to yr="2014 --Current"
69	limit 66 to yr="2011 --Current"

The Cochrane Central Register of Controlled Trials (CENTRAL)

#1	MeSH descriptor: [Child Development Disorders, Pervasive] explode all trees 688
#2	ASD:ti,ab
#3	Asperger*:ti,ab
#4	PDD:ti,ab
#5	autistic:ti,ab
#6	autism:ti,ab
#7	(#1 or #2 or #3 or #4 or #5 or #6)
#8	invention:ti,ab
#9	treat*:ti,ab
#10	therap*:ti,ab
#11	MeSH descriptor: [Communication] explode all trees
#12	communication*:ti,ab
#13	interpersonal*:ti,ab
#14	speech*:ti,ab
#15	interact*:ti,ab
#16	synchron*:ti,ab
#17	relationship*:ti,ab
#18	language*:ti,ab
#19	social:ti,ab

#20 behaviour*:ti,ab
#21 behavior*:ti,ab 25930
#22 MeSH descriptor: [Behavior] explode all trees
#23 MeSH descriptor: [Behavior Therapy] explode all trees
#24 "behavior therap*":ti,ab
#25 ABA:ti,ab
#26 modification*:ti,ab
#27 (#8 or #9 or #10 or #11 or #12 or #13 or #14 or #15 or #16 or #17 or #18 or #19 or #20 or #21 or #22 or #23 or #24 or #25 or #26)
#28 #7 and #27
#29 MeSH descriptor: [Child, Preschool] this term only
#30 child*:ti,ab
#31 preschool*:ti,ab
#32 MeSH descriptor: [Schools, Nursery] this term only
#33 nurser*:ti,ab
#34 kindergarten*:ti,ab
#35 MeSH descriptor: [Infant] this term only
#36 infant*:ti,ab
#37 baby:ti,ab
#38 babies:ti,ab
#39 toddler*:ti,ab
#40 (#29 or #30 or #31 or #32 or #33 or #34 or #35 or #37 or #38 or #39)
#41 (#28 and #40)
#42 MeSH descriptor: [Animals] explode all trees
#43 MeSH descriptor: [Humans] this term only
#44 #42 not (#42 and #43)
#45 #41 not 44
#46 #45 in Trials
#47 #45 Publication Year to 2014, in Trials

ERIC
"S1 DE ""Pervasive Developmental Disorders"" OR DE ""Asperger Syndrome"" OR DE ""Autism"""
S2 TI(autism) OR AB(autism)
S3 TI(autistic) OR AB(autistic)
"S4 TI(""ASD"") OR AB(""ASD"") "
S5 TI(asperger*) OR AB(asperger*)
"S6 TI(""Pervasive Developmental Disorder*"") OR AB(""Pervasive Developmental Disorder*"") "
"S7 TI(""PDDNOS"") OR AB(""PDDNOS"") "
"S8 TI(""PDD-NOS"") OR AB(""PDD-NOS"") "
S9 S1 OR S2 OR S3 OR S4 OR S5 OR S6 OR S7 OR S8
S10 TI(intervention*) OR AB(intervention*)
"S11 DE ""Intervention"" OR DE ""Crisis Intervention"" OR DE ""Early Intervention"" OR DE ""Prereferral Intervention"" OR DE ""Response to Intervention"" OR DE ""School Turnaround"""
S12 TI(interpersonal*) OR AB(interpersonal*)
"S13 DE ""Speech"" OR DE ""Articulation (Speech)"" OR DE ""Artificial Speech"" OR DE ""Inner Speech (Subvocal)"" OR DE ""Pronunciation"" OR DE ""Speech Acts"""
S14 TI (speech) OR AB(speech)
S15 TI(interact*) OR AB(interact*)
S16 TI(synchron*) OR AB(synchron*)
S17 TI(relationship*) OR AB(relationship*)
"S18 DE ""Relationship"" OR DE ""Community Relations"" OR DE ""Family School Relationship"" OR DE ""Parent School Relationship"" OR DE ""Family Work Relationship"" OR DE ""Human Relations"" OR DE ""Interaction"" OR DE ""Aptitude

Treatment Interaction"" OR DE ""Feedback (Response)"" OR DE ""Group Dynamics"" OR DE ""Interpersonal Relationship"" OR DE ""Caregiver Child Relationship"" OR DE ""Counselor Client Relationship"" OR DE ""Dating (Social)"" OR DE ""Family

Relationship"" OR DE ""Friendship"" OR DE Patient Relationship"" OR DE ""Supervisor Supervisee Relationship"" OR DE ""Teacher Student Relationship"" OR DE ""Intimacy"" OR DE ""Regular and Special Education Relationship""""Group Unity""

OR DE ""Helping Relationship"" OR DE ""Interpersonal Attraction"" OR DE ""Inter professional Relationship"" OR DE ""Parent Caregiver Relationship"" OR DE ""Peer Relationship"" OR DE ""Physician"
"S19 DE ""Behavior"" OR DE ""Adjustment (to Environment)"" OR DE ""Affective Behavior"" OR DE ""Assertiveness"" OR DE ""Attachment Behavior"" OR DE ""Child Behavior"" OR DE ""Infant Behavior"" OR DE ""Competition"" OR DE

""Cooperation"" OR DE ""Group Behavior"" OR DE ""Health Behavior"" OR DE ""Hyperactivity"" OR DE ""Imitation"" OR DE ""Life Style"" OR DE ""Modeling

(Psychology)"" OR DE ""Parenting Styles"" OR DE ""Participation"" OR DE ""Audience

Participation"" OR DE ""Community Involvement"" OR DE ""Family Involvement"" OR DE ""Parent Participation"" OR DE ""School Involvement"" OR DE ""Student Participation"" OR DE ""Teacher Participation"" OR DE ""Performance"" OR DE

""Counselor Performance"" OR DE ""Failure"" OR DE ""Success"" OR DE ""Persistence"" OR DE ""Academic Persistence"" OR DE ""Teacher Persistence"" DE ""Physical Activity Level"" OR DE ""Resistance (Psychology)"" OR DE ""Resistance to

Change"" OR DE ""Response Style (Tests)"" OR DE ""Responses"" OR DE ""Audience Response"" OR DE ""Burnout"" OR DE ""Dimensional Preference"" OR DE ""Emotional Response"" OR DE ""Motor Reactions"" OR DE ""Patterned Responses"" OR DE

""Stranger Reactions"" OR DE ""Student Reaction"" OR DE ""Teacher Response"" OR DE ""Self Control"" OR DE ""Social Behavior"" OR DE ""Student Behavior"" OR DE ""Student Adjustment"" OR DE ""Student Participation"" OR DE ""Student

Reaction"" DE ""Teacher Behavior"" Behavior"" OR DE ""Health Behavior"" OR DE ""Hyperactivity"" OR DE ""Imitation"" OR DE ""Life Style"" OR DE ""Modeling (Psychology)"" OR DE ""Parenting Styles"" OR DE ""Participation"" OR DE ""Audience

Participation"" OR DE ""Community Involvement"" OR DE ""Family Involvement"" OR DE ""Parent Participation"" OR DE ""School Involvement"" OR DE ""Student Participation"" OR DE ""Teacher Participation"" OR DE ""Performance"" OR DE

""Counselor Performance"" OR DE ""Failure"" OR DE ""Success"" OR DE ""Persistence"" OR DE ""Academic Persistence"" OR DE ""Teacher Persistence"" DE ""Physical Activity Level"" OR DE ""Resistance (Psychology)"" OR DE ""Resistance to

Change"" OR DE ""Response Style (Tests)"" OR DE ""Responses"" OR DE ""Audience Response"" OR DE ""Burnout"" OR DE ""Dimensional Preference"" OR DE ""Emotional Response"" OR DE ""Motor Reactions"" OR DE ""Patterned Responses"" OR DE

""Stranger Reactions"" OR DE ""Student Reaction"" OR DE ""Teacher Response"" OR DE ""Self Control"" OR DE ""Social Behavior"" OR DE ""Student Behavior"" OR DE ""Student Adjustment"" OR DE ""Student Participation"" OR DE ""Student

Reaction"" DE ""Teacher Behavior"" "
S20 TI(behavior*) OR AB(behavior*)
S21 TI(behaviour*) OR AB(behaviour*)

S22 TI(treat*) OR AB(treat*)
"S23 DE ""Therapy"" OR DE ""Art Therapy"" OR DE ""Bibliotherapy"" OR DE ""Drug Therapy"" OR DE ""Educational Therapy"" OR DE ""Group Therapy"" OR DE ""Hearing Therapy"" OR DE ""Music Therapy"" OR DE ""Occupational Therapy"" OR DE

""Physical Therapy"" OR DE ""Psychotherapy"" OR DE ""Speech Therapy"" OR DE ""Therapeutic Recreation"""
S24 TI(therap*) OR AB(therap*)
"S25 TI(""ABA"") OR AB(""ABA"") "
S26 TI(social) OR AB(social)
"S27 DE ""Communication (Thought Transfer)"" OR DE ""Classroom Communication"" OR DE ""Computer Mediated Communication"" OR DE ""Discussion"" OR DE ""Group Discussion""OR DE ""Intercultural Communication"" OR DE ""Interpersonal

Communication"" OR DE ""Nonverbal Communication"" OR DE ""Organizational Communication"" OR DE ""Interschool Communication"" OR DE ""Speech Communication"" OR ""Public speaking"" OR DE ""Verbal Communication"""
"S28 DE ""Language"" OR DE ""Artificial Languages"" OR DE ""Child Language"" OR DE ""Interlanguage"" OR DE ""Language of Instruction"" OR DE ""Languages for Special Purposes"" OR DE ""Native Language"" OR DE ""Oral Language"" OR DE

""Second Languages"" OR DE ""English (Second OR DE ""Graphemes"" OR DE ""Ideography"" OR DE ""Orthographic Symbols"" OR DE ""Punctuation""Language)""OR DE ""Sign Language"" OR DE ""American Sign Language"" OR DE ""Symbolic Language""

OR DE ""Written Language"" OR DE ""Braille"""
S29 TI(language*) OR AB(language*)
"S30 (DE ""Development"" OR DE ""Capacity Building"" OR DE ""Educational Development"" OR DE ""Curriculum Development"" OR DE ""Instructional Development"" OR DE ""Individual Development"" OR DE ""Adolescent Development"" OR DE ""Aging

(Individuals)"" OR DE ""Behavior Development"" OR DE ""Career Development"" OR DE ""Child Development"" OR DE ""Cognitive Development"" OR DE ""Creative Development"" OR DE ""Emotional Development"" OR DE ""Moral Development"" OR DE

""Personality Development"" OR DE ""Physical Development"" OR DE ""Skill Development"" OR DE ""Social Development"" OR DE ""Spiritual Development"" OR DE ""Talent Development"" OR DE ""Material Development"" OR DE ""Program

Development"" OR DE ""Student Development"" OR DE ""Vocabulary Development"")"
S31 TI(development*) OR AB(development*)

S32 TI(modification*)   OR AB(modification*)
S33 TI Communication*   OR AB Communication*
"S34 DE ""Behavior Modification""   "
S35 S10 OR S11 OR S12 OR S13 OR S14OR S15 OR S16 OR S17 OR S18 OR S19 OR S20 OR S21 OR S22 OR S23 OR S24 OR S25 OR S26 OR S27 OR S28 OR S29 OR S30 OR S31 OR S32 OR S33 OR S34
S36 TI(child*) OR AB(child*)
S37 TI(baby) OR AB(baby)
S38 TI(babies) OR AB(babies)
S39 TI(infan*) OR AB(infan*)
S40 TI(preschool) OR AB(preschool)
S41 TI(kindergarten*)   OR AB(kindergarten*)
"S42   DE ""Children"" OR DE ""African American Children"" OR DE ""Grandchildren""   OR DE ""Hospitalized Children"" OR DE ""Latchkey Children"" OR DE ""Migrant Children"" OR DE ""Minority Group Children"" OR DE ""Preadolescents""   OR DE

""Young Children"""
"S43 DE ""Infants"" OR DE ""Preschool Children"" OR DE ""Toddlers"""
"S44 DE ""Neonates"" OR DE ""Premature Infants"""
"S45 DE ""Kindergarten""   "
"S46 DE ""Nursery Schools""   "
S47 TI(nurser*) OR AB(nurser*)
S48 TI(Toddler*) OR AB(Toddler*)
S49 S36 OR S37 OR S38 OR S39 OR S40 OR S41 OR S42 OR S43 OR S44 OR S45 OR S46 OR S47 OR S48
S50 S9 AND S35
S51 S49 AND S50
S52 AB(randomized)
S53 AB(placebo)
S54 AB(randomly)
S55 AB(trial)
S56 AB(groups)
S57 S52 OR S53 OR S54 OR S55 OR S56
S58 S51 AND S57

CINAHL

S1 (MH "Child Development Disorders, Pervasive+")
S2 TI(autism) OR AB(autism)
S3 TI(autistic) OR AB(autistic)
S4 TI("ASD") OR AB("ASD")
S5 TI(asperger*) OR AB(asperger*)
S6 TI("Pervasive Developmental Disorder*") OR AB("Pervasive Developmental Disorder*")
S7 TI("PDDNOS") OR AB("PDDNOS")

S8 TI("PDD-NOS") OR AB("PDD-NOS")

S9 S1 OR S2 OR S3 OR S4 OR S5 OR S6 OR S7 OR S8
S10 TI(intervention*) OR AB(intervention*)
S11 TI(interpersonal*) OR AB(interpersonal*)

S12 (MH "Speech")
S13 TI (speech) OR AB(speech)
S14 TI(interact*) OR AB(interact*)

S15 TI(synchron*) OR AB(synchron*)
S16 TI(relationship*) OR AB(relationship*)
S17 TI(behavior*) OR AB(behavior*)
S18 (MH "Child Behavior+")
S19 (MH "Behavior+")
S20 (MH "Behavior Modification+")
S21 TI(treat*) OR AB(treat*)
S22 TI(therap*) OR AB(therap*)

S23 TI(modification*) OR AB(modification*)
S24 TI("ABA") OR AB("ABA")
S25 TI(behaviour*) OR AB (behaviour*)
S26 TI(social) OR AB(social)
S27 TI(communication*) OR AB(communication*)
S28 TI(language*) OR AB(language*)
S29 S10 OR S11 OR S12 OR S13 OR S14 OR S15 OR S16 OR S17 OR S18 OR S19 OR S20 OR S21 OR S22 OR S23 OR S24 OR S25 OR S26 OR S27 OR S28
S30 (MH "Child+")
S31 TI(baby) OR AB(baby)
S32 TI(babies) OR AB(babies)
S33 TI(infan*) OR AB(infan*)
S34 TI(preschool*) OR AB(preschool*)
S35 TI(child*) OR AB(child*)
S36 TI(kindergarten*) OR AB(kindergarten*)
S37 (MH "Schools, Nursery")

S38 TI(nursery school*) OR AB(nursery school*)
S39 S30 OR S31 OR S32 OR S33 OR S34 OR S35 OR S36 OR S37 OR S38
S40 (singl* blind*)
S41 (doubl* blind*)
S42 (tripl* blind*)
S43 (trebl* blind*)
S44 (trebl* mask*)
S45 (tripl* mask*)
S46 (doubl* mask*)
S47 (singl* mask*)
S48 (Randomi?ed control*
S49 (MH “Random
S50 random* allocat*
S51 placebo*
S52 (MH “Placebos”)
S53 (MH “Quantitative
S54 allocat* random*
S55 S40 OR S41 OR S42 OR S43 OR S44 OR S45 OR S46 OR S47 OR S48 OR S49 OR S50 OR S51 OR S52 OR S53 OR S54
S56 S9 AND S29 AND S39
S57 S55 AND S56
S58 S55 AND S56
S59 S55 AND S56
S60 S55 AND S56
S61 S55 AND S56
S62 S55 AND S56
