# Supplementary material for: Meta-analyses of individual versus group interventions for pre-school children with autism spectrum disorder (ASD)
Source: PLoS One. 2018 May 15;13(5):e0196272. doi: 10.1371/journal.pone.0196272 (PMC5953451; doi:10.1371/journal.pone.0196272)
Supplement: S1 Table — (PDF) [file pone.0196272.s004.pdf]

S1 Table. Characteristics of the included studies.

Studies	Country	no. of participants; (intervention group) vs (control group) (gender; Male: Female)	Age (years, range)	Intervention Program	Intervention type	Intervention and dose	Comparison	Duration (weeks)
Aldred 2004	UK	N-=28; (n=14) vs (n=14); (25 M: 3 F)	2;0-5:11 years	Social communication intervention	Individual	Monthly treatment session Between the sessions, families were also asked to do 30 min of daily home practice with routine care.Monthly treatment session Between the sessions, families were also asked to do 30 min of daily home practice with routine care.	Received routine care alone	12 months
Carter 2011	USA	N=62; (n=32) vs (n=30); (51 M: 11 F)	1;3-2;3 years	Hanen's More Than Words	Individual	Eight group sessions for parents, and three in-home parent-child sessions.	Business as usual	3.5 months
Casenheiser 2011	Canada	N=51; (n=25)vs(n=26)	2;0-4;11 years	Social-communication-based intervention	Individual	2 hours of therapy and coaching every week, and approximately 3 hours per day spent interacting with their children	Community treatment (various treatment no more than 15 hours per week)	12 months
Dawson 2010	USA	N=45 (n=24)vs(n=21); (35 M :10 F) N=48 were allocated to the trials (n=24 vs n=24)	1;2-2;6 years	Early Start Denver Model	Individual	2 hour sessions twice a day, 5 days a week	Referral to community providers for intervention commonly available in the community.	2 years
Drew 2002	UK	N=24 (n=12)vs(n=12); (19 M: 5 F)	22.5 (SD = 3.4) months	Parent training intervention with a focus on the development of joint attention skills and joint action routines	Individual	3 hours parents session every 6 weeks	Receiving locally available services only	12 weeks
Goods 2013	USA	N=15 (n=6)vs(=5)	3-5 years	JASPER (Joint Attention Symbolic Play Engagement and Regulation) program	Group	The number of the children in the intervention group was 7 (2 children withdrew). 30 minutes (as a substitute to ABA) intervention twice weekly during children's regular program, 24 sessions over 12 weeks	Treatment as usual in local service	12 weeks

Green 2010	UK	N=152 (n=77) vs (n=75); (138 M; 14 F)	2;0-4;11 years	Preschool Autism Communication Trail (PACT)	Individual	Biweekly 2 h clinic sessions for 6 months followed by monthly booster sessions for 6 months (total 18). Between the sessions, families were also asked to do 30 min of daily home practice. biweekly 2 h clinic sessions for 6 months followed by monthly booster sessions for 6 months (total 18). Between the sessions, families were also asked to do 30 min of daily home practice.	Treatment as usual	12 months (13 month)
Ichikawa 2013	Japan	N=11 (n=5)vs(n=6); (9 M: 2 F)	5-6 years	TEACCH-based group social skills training for children with ASD and their mothers	Group	The number of the children in the intervention group was 5. 2-hour sessions weekly (20 sessions in total)	No therapy (waitlist for intervention)	6 months
Ingersoll 2010	USA	N=22 (n=11) vs (n=11)	2;3-3;11 years	Reciprocal Imitation Training for Teaching Elicited and Spontaneous Imitation to Children with Autism	Individual	1 hour per day, 3 days a week	Treatment as usual in the community	10 weeks
Ingersoll 2012	USA	N=29 (n=15) vs (n=14)	2;3-3;11 years	Reciprocal Imitation Training for Teaching Elicited and Spontaneous Imitation to Children with Autism	Individual	1 hour per day, 3 days a week	Treatment as usual in the community	10 weeks
Jocelyn 1998	Canada	N = 35 (n=16) vs (n=19); (34 M: 1 F)	3-5 years (Experimental group 42.6±9.2 month, control group 43.8±9.0 month)	Caregiver-based intervention program in community day-care centers	Individual	Hospital-based seminar (3 hours, 5 days a week) and on-site consultations with the autism behaviour specialist (3 hours per week for 10 weeks)	Treatment as usual in the community day-care centers	12 weeks
Kaale 2012	Norway	N=61 (n=34) vs (n=27); (48 M: 13 F)	2;5-5 years	Preschool-based joint attention intervention for children with autism	Group	The intervention program was performed in the mainstream preschools, ASD-units in mainstream preschools, and ASD preschools. Two daily interventions 5 days a week (80 sessions were aimed). 20 minutes session consists of 5 min. of table top training for targeted JA skills, 15 min. of floor play for generalization of the skills	Receiving ordinary preschool program only	8 weeks
Kaale 2014				Follow-up study of Kaale 2012	Group			

Kasari 2010	USA	N=38 (n=19) vs (n=19); (29 M: 9 F)	1;7-3 years	Caregiver Mediated Joint Engagement Intervention for Toddlers with Autism	Individual	24 therapist sessions with care and child, delivered at 3 sessions per week with each session lasting about 45 minutes.	No therapy (wait list for intervention)	8 weeks (14 months)8 weeks (14 months)
Kim 2008	Korea	N=15 (n=5) vs (n=5) (13 M: 2 F)	3-5 years (Mean 51.20 months, SD 12.08 month)	Improvisational music therapy	Individual	Weekly 30 minute sessions for 12 consecutive weeks	control condition of play sessions with toys.	12 weeks
Kim 2009				Sub-analyses paper of Kim 2008	Individual			
Landa 2011	USA	N=50 (n=25) vs (n=25); (40 M: F 10)	1;9-2;9 years	Intervention targeting development of socially synchronous engagement in toddlers with ASD	Group	10 hours per week in classroom, home-based parent training (1.5 hours per month) and parent education (38 hours)	Non supplementing intervention to a curriculum targeting socially synchronous behavior on social outcomes of toddlers with autism spectrum disorders	6 months
Lawton 2012	USA	N=16 (n=9) vs (n=7)	3-5 years	The Joint Attention and Symbolic Play/Engagement and Regulation intervention (JASP/ER)	Group	Teacher-implemented joint attention intervention for 30 minutes two times a week (10 times) every day at preschool classroom	Wait list for treatment	6 week
Pajareya 2011	Thailand	32 (n=16) vs (n=16); (28 M: F4)	2-6 years	Home-based Developmental, Individual-Difference, Relationship-Based (DIR)/Floortime intervention	Individual	15.2 hours per week (expected 20 hours per week)	The typical treatment	3 months
Reitzel 2013	Canada	15 (n=8) vs (n=7)	38-82 months (Mean=58.5 months; SD=14.3)	Functional Behavior Skills Training (FBST)	Individual	2-hours group sessions per week (a 30-min parents-only training session, a simultaneous children's activity session, and a 90-min combined children's and parents' training session)	Treatment as usual	4 months
Roberts 2011 Home-based program	Australia	Total n = 87 (Home-based + Center-based + control group) (90.5% were male) (n = 28) vs (n = 29)	26.5-60.3 months (Mean 41.5 months, range 26.5-59.4 months) vs (Mean 43.7 months, range 27.6-60.3 months)	Building Blocks programme's an individualised home-based service	Individual	Visits for 2 h once a fortnight by stuff of the trans-disciplinary team	Wait list for treatment	Over a 40 week period (20 session maximum)
Roberts 2011 Centre-based program	Australia	Total n = 87 (Home-based + Center-based + control group) (90.5% were male) (n = 28) vs (n = 29)	26.3-60.3 months (Mean 41.5 months, range 26.5-59.4 months) vs (Mean 43.7 months, range 27.6-60.3 months)	Building Blocks programme's small group centre-based service for children combined with a parent training and support group	Group	six playgroups of 4-6 children, 40 weekly 2h sessions, with six concurrent parent support and training groups	Wait list for treatment	40 weekly 2 h sessions

Rogers 2012	USA	98 (n=49) vs (n=49); (76 M: F 22)	1;2-2;0 years	Parent delivery of the Early Start Denver Model (P-ESDM)	Inidividual	12 consecutive sessions, each session lasting 1 hour	Community treatment as usual.	12 weeks
Schertz 2013	USA	23 (n=11) vs (n=12)	under 2;6 years (mean age of the intervention group: 24.6 months, control group: 27.5 months)	Joint Attention Mediated Learning (JAML) intervention only	Inidividual	Weekly at home for at least 15 sessions, Parents agree to spend 30 minutes per day in face-to-face interaction with their child.	No JAML intervention during study except general early intervention services and some specialized ASD-related interventions such as intensive applied behavior analysis.	4-12 months (mean 7 months)
Siller 2013	USA	70 (n=36)vs (n=34); (64 M: 6 F)	Mean age of the control group: 58.3 months (SD = 12.7), Control group: 55.9 months (SD 11.9)	Focused Playtime Intervention (FPI) (Parent-mediated intervention to increase responsive parental behaviors and child communication in children with ASD)	Inidividual	12 in-home training sessions (1 session per week, 90 minutes per session)	Four sessions for Parent Advocacy Coaching, PAC, without FPI intervention.	12 weeks
Siller 2014				Same as Siller 2013	Inidividual			
Smith 2000	USA	28 (n=15) vs (n=13); (23 M: 5 F)	Mean age of the control group: 36.07 months (SD = 6.00), Control group: 35.77 months (SD 5.37)	Intensive treatment which was defined as 30 hours per week of intervention for each child for 2 to 3 years	Individual	24.52 hours per week of individual treatment for one year, gradually reducing hours over the next 1 to 2 years.	Parents training	2 years
Thompson 2012	Australia	N=23 (n=12) vs (n=11) (M 19: 4 F)	3 to 6 years	Home-based , family-centered music therapy (using songs, improvisation, structured interactions) with standard care	Individual	Family- centered music therapy plus standard care	Waitlist with no therapy	16 weeks
Tonge 2006	Australia	105 (n=35; n=35) vs (n=35)	2.5-5 years Mean age of the control group: 35.2 months (SD = 3.8), Control group: 33.3 months (SD 4.2)	Education and Skills Training Program forParensts of Young children with autism PEBM Skills training intervention or control for the non specific aspects of the PEBM (PEC) intervention	Individual	Ten 60-min individual family sessions and ten 90-min small group (4-5 families) sessions over a 20-week period.	Received local early childhood services only but no PEBM and PEC interventions	20 week (12 month)
Tonge 2014				Same as Tonge 2006	Individual			

Venker 2011	USA	14 (n=7) vs (n=7)	2;4-5;8 years Mean 41.14 months (SD10.40 months)	Interventions for increasing verbal responsiveness in parents of children with Autism	Individual	Total eight to 10 hours of parent education, 1.5 hours of individual sessions with the Hanen-certified speech language pathologist, and 12 to 14 hours of small group sessions.	Waitlist	6 week
Welterlin 2012	USA	20 (n=10) vs (n=10); (18 M: 2 F)	2 to 3 years Mean age of the control group: 30.5 months (SD = 3.6), Control group: 30.5 months (SD 4.3)	HomeTEACCHing Program for Toddlers with autism	Individual	1.5 hours a week for 12 sessions	Waitlist	12 weeks
