# Supplementary material for: Meta-analyses of individual versus group interventions for pre-school children with autism spectrum disorder (ASD)
Source: PLoS One. 2018 May 15;13(5):e0196272. doi: 10.1371/journal.pone.0196272 (PMC5953451; doi:10.1371/journal.pone.0196272)
Supplement: S2 Table — (PDF) [file pone.0196272.s005.pdf]

**S2 Table. Outcome measure list used in the included studies for the data syntheses.**

| | | | Pre (T1) | | Pre (T1) | | post (T2) | | | Post (T2) | | |
|---|---|---|---|---|---|---|---|---|---|---|---|---|
| | | | Experiment | | Control | | Experiment | | | Control | | |
| | Outcome | Measures | post-treatment outcome | | Post-treatment outcome | | post-treatment outcome | | | Post-treatment outcome | | |
| | | | Mean | SD | Mean | SD | Mean | SD | n | Mean | SD | n |
| Aldred et al. (2004) | Autism symptoms: general | ADOS, total | 16.1 | 4.5 | 15.6 | 4.9 | 11.8 | 6.4 | 14 | 16.1 | 4.4 | 14 |
| | Expressive language | MCDI, expressive language | 28.0 | 467.0 | 25.6 | 683.0 | 199.4 | 25606.0 | 14 | 33.1 | 683.0 | 14 |
| | Receptive language | MCDI, language comprehension | 71.7 | 2.4 | 95.4 | 426.0 | 222.7 | 40431.0 | 14 | 146.8 | 11426.0 | 14 |
| | Maternal synchronization | Parent synchrony | 57.8 | 15.0 | 57.1 | 49.5 | 65.1 | 14.3 | 14 | 49.5 | 18.9 | 14 |
| | Joint engagement | Child communication acts | 30.8 | 10.2 | 30.1 | 11.1 | 37.6 | 10.1 | 14 | 27.6 | 16.5 | 14 |
| Carter et al. (2011) | Receptive language | MSEL, receptive Language | 8.41 | 5.42 | 8.17 | 4.44 | 15.52 | 6.93 | 25 | 17.48 | 8.33 | 25 |
| | Expressive language | MSEL, expressive Language | 8.22 | 6.01 | 7.33 | 3.71 | 16.2 | 7.23 | 25 | 16.68 | 7.88 | 25 |
| | Developmental quotient | MSEL, composite score | | | | | 63.88 | 18.41 | 25 | 64.88 | 13.94 | 25 |
| | Initiating joint attention | ESCS, frequency of initiating joint attention | 5.90 | 5.41 | 5.59 | 6.14 | 10.33 | 9.82 | 24 | 8.68 | 9.26 | 25 |
| | Maternal synchronization | PCFP, proportion of codable intervals with parental responsivity | 0.32 | 0.06 | 0.29 | 0.08 | 0.34 | 0.07 | 23 | 0.3 | 0.10 | 24 |
| Casenhiser et al. (2011) | Initiating joint attention | Modified CBRS, initiation of joint attention | 1.28 | 0.54 | 1.31 | 0.99 | 1.84 | 0.55 | 16 | 1.23 | 0.43 | 13 |
| Dawson et al. (2010) baseline imbalance | Developmental quotient | MSEL, early learning composite | 76.4 | 23.4 | 64.0 | 13.8 | 78.6 | 24.2 | 24 | 66.3 | 15.3 | 21 |
| | Receptive language | MSEL, receptive language | 38.9 | 15.4 | 31.1 | 11.1 | 40 | 16.3 | 24 | 31.5 | 10.6 | 21 |
| | Expressive language | MSEL, expressive language | 36.1 | 14.2 | 33.0 | 11.5 | 36.6 | 13.6 | 24 | 30 | 9.2 | 21 |
| | Adaptive behavior | VABS, composite | 65.7 | 9.8 | 63.7 | 8.8 | 68.7 | 15.9 | 24 | 59.1 | 8.8 | 21 |
| | Autism symptoms: general | ADOS, severity score | 6.5 | 1.5 | 7.3 | 2.1 | 7.0 | 1.9 | 24 | 7.3 | 1.8 | 21 |
| | Autism symptoms: restricted repetitive and stereotyped patterns of behavior, interests, and activities | RBS | 15.5 | 12.3 | 23.3 | 17.5 | 16.7 | 13.1 | 24 | 22 | 16.3 | 21 |
| Drew et al. (2002) | Developmental quotient | Non-verbal IQ from D and E scales of GSMD | 88.1 | 11.2 | 66.0 | 16.5 | 77.9 | 14.8 | 12 | 66.1 | 17.1 | 12 |
| | Receptive language | MCDI, words understood | 52.0 | 60.5 | 53.0 | 63.7 | 176.1 | 121.9 | 12 | 100.3 | 80.2 | 12 |
| | Expressive language | MCDI, words said | 6.8 | 20.9 | 6.6 | 13.7 | 96.6 | 118.8 | 12 | 44 | 50.2 | 12 |
| | Autism symptoms: qualitative impairments in social | ADI, reciprocal social interaction | 19.6 | 3.0 | 20.3 | 4.5 | 18.3 | 4.9 | 12 | 20.1 | 4.3 | 12 |
| | Autism symptoms: qualitative impairments in communication | ADI, nonverbal communication | 12.8 | 1.6 | 12.0 | 2.4 | 11 | 2.8 | 12 | 11.9 | 1.8 | 12 |

| | | | | | | | | | | | | |
|---|---|---|---|---|---|---|---|---|---|---|---|---|
| | Autism symptoms: restricted repetitve and stereotyped patterns of behavior, interests, and activities | ADI, repetitive and stereotyped behavior | 3.2 | 1.1 | 3.7 | 1.6 | 3.9 | 1.8 | 12 | 4.2 | 2 | 12 |
| | Parental stress | PSInv., total score | 113.8 | 21.7 | 110.0 | 28.6 | 104.3 | 20 | 12 | 112.1 | 20.1 | 12 |
| Goods et al. (2013) | Functional play | Structured Play Assessment, play types | 21.14 | 7.58 | 17.13 | 6.83 | 22.00 | 10.17 | 5 | 14.33 | 9.69 | 6 |
| | Initiating joint attention | ESCS, initiating joint attention | 2.57 | 4.39 | 0.75 | 2.12 | 0.40 | 1.89 | 5 | 1 | 1.73 | 6 |
| | Expressive language | RDLS, expressive language | 13.63 | 4.57 | 11.93 | 0.09 | 14.52 | 5.38 | 5 | 11.95 | 0.16 | 6 |
| | Receptive language | RDLS, verbal comprehension | 12.14 | 0.41 | 12.00 | 0.34 | 14.59 | 5.36 | 5 | 12.05 | 0.38 | 6 |
| Green et al. (2010) | Autism general symptom | ADOS-G, total social-communication algorithm score | 19.6 | 4.2 | 19.3 | 4.0 | 15.7 | 6.0 | 77 | 16.5 | 5.7 | 75 |
| | Autism symptoms: qualitative impairments in social interaction | ADOS-G, social domain | 10.7 | 7.2 | 10.7 | 2.1 | 9.2 | 3.0 | 77 | 9.8 | 2.9 | 75 |
| | Autism symptoms: qualitative impairments in communication | ADOS-G, communication domain | 8.9 | 2.5 | 8.6 | 2.5 | 6.6 | 3.3 | 77 | 6.7 | 3.2 | 75 |
| | Autism symptoms: restricted repetitve and stereotyped patterns of behavior, interests, and activities | ADOS-G, repetitive-behaviors | 3.7 | 1.5 | 3.7 | 1.4 | 3.0 | 1.7 | 77 | 3.5 | 1.6 | 75 |
| | Receptive language | PLS, receptive raw scores | 15.6 | 9.8 | 15.0 | 9.7 | 21.5 | 13.0 | 77 | 20.3 | 12.8 | 75 |
| | Expressive language | PLS, expressive raw scores | 15.0 | 8.1 | 15.1 | 7.9 | 20.0 | 11.2 | 77 | 20 | 11.3 | 75 |
| | Maternal synchronization | Parent-child interaction, parental synchrony | 0.3 | 14.8 | 0.3 | 14.6 | 0.5 | 19.6 | 77 | 0.33 | 14 | 75 |
| | Joint engagement | Parent-child interaction, child initiation | 0.2 | 17.4 | 0.2 | 18.5 | 0.3 | 19.7 | 77 | 0.26 | 17.5 | 75 |
| Ichikawa et al. (2013) | Parenting stress | PSInd. | 226.2 | 31.5 | 198.2 | 26.8 | 213.0 | 34.8 | 5 | 204.3 | 36.3 | 6 |
| | N/A | BDI | 16.0 | 7.7 | 9.8 | 5.3 | 12.8 | 10.3 | 5 | 8.5 | 4.8 | 6 |
| | Aadaptive behavior | SDQ | 19.0 | 3.5 | 13.2 | 3.3 | 14.4 | 4.7 | 5 | | 12.5 | 6 |
| | Joint engagement | IRS | 38.9 | 4.8 | 41.5 | 3.0 | 40.2 | 5.1 | 5 | 39.7 | 6.0 | 6 |
| Ingersoll (2010) | Imitation | UIA | | | | | 14.93 | 11.54 | 11 | 4.92 | 4.70 | 10 |
| Ingersoll (2012) | Initiating joint attention | ESCS: initiating joint attention | | | | | 5.93 | 6.72 | 14 | 1.46 | 2.11 | 13 |
| | Adaptive behavior | SES | | | | | 101.14 | 27.98 | 14 | 91.08 | 18.73 | 13 |
| Kaale et al. (2012) | Initiating joint attention | Joint attention during performing ESCS | 1.3 | 2.8 | 1.3 | 1.8 | 1.6 | 2.6 | 33 | 1.6 | 2.2 | 27 |
| | Joint engagement | Joint engagement during mother-child play (%) | 45.1 | 23.4 | 50.2 | 21.7 | 57.3 | 22.8 | 34 | 49.2 | 19.9 | 27 |
| Kaale et al. (2014) | Initiating joint attention | Frequency of child initiation of joint attention during performing ESCS | 5.7 | 6.7 | 5.9 | 6.7 | 8.1 | 6.87 | 34 | 6.2 | 6.9 | 27 |
| | Receptive language | RDLS, receptive language | 20.0 | 15.0 | 25.1 | 16.3 | 32.8 | 10.14 | 32 | 39.4 | 10.1 | 27 |
| | Expressive language | RDLS, expressive language | 14.2 | 14.2 | 20.1 | 17.0 | 27.9 | 10.68 | 32 | 34.1 | 10.7 | 27 |

| | | | | | | | | | | | | |
|---|---|---|---|---|---|---|---|---|---|---|---|---|
| | Autism symptoms: qualitative impairment in social interaction | SCQ: C, social subscale, parents | | | | | 5.3 | 3.85 | 29 | 4.1 | 3.9 | 23 |
| | Autism symptoms: qualitative impairment in communication | SCQ: C, communication subscale, parents | | | | | 4.1 | 2.16 | 29 | 4.5 | 2.2 | 23 |
| Kasari et al. (2010) | Joint engagement | Joint engagement | 30.26 | 14.91 | 24.98 | 10.74 | 42.85 | 19.96 | 19 | 27.87 | 14.01 | 19 |
| | Initiating joint attention | Frequency of joint attention initiation | 3.00 | 2.77 | 3.62 | 5.92 | 3.11 | 3.41 | 19 | 3.77 | 3.76 | 19 |
| | Responding to joint attention | Frequency of joint attention responses | 0.42 | 0.69 | 0.63 | 0.23 | 0.79 | 0.23 | 19 | 0.05 | 0.23 | 19 |
| | Functional play | Type of functional play acts | 3.00 | 2.38 | 5.29 | 2.37 | 5.29 | 2.37 | 19 | 3.29 | 2.30 | 19 |
| | Symbolic play | Type of symbolic play acts | 0.11 | 0.46 | 0.42 | 0.84 | 0.26 | 0.65 | 19 | 0.53 | 1.43 | 19 |
| Kim et al. (2008) | Initiating joint attention | ESCS, initiation joint attention | 2.4 | 3.78 | 5.8 | 4.87 | 5.8 | 6.22 | 5 | 9 | 6.28 | 5 |
| | Responding to joint attention | ESCS, responding to joint attention | 5.2 | 5.59 | 7.2 | 4.82 | 7.2 | 6.46 | 5 | 9.4 | 3.78 | 5 |
| Landa et al. (2011) | Imitation | Socially engaged imitation | 0.17 | 0.19 | 0.25 | 0.24 | 0.44 | 0.22 | 24 | 0.28 | 0.21 | 24 |
| | Initiating joint attention | ESCS, initiation joint attention | 2.4 | 3.78 | 5.8 | 4.87 | 5.8 | 6.22 | 24 | 9 | 6.28 | 24 |
| | Responding to joint attention | ESCS, responding to joint attention | 5.2 | 5.59 | 7.2 | 4.82 | 7.2 | 6.46 | 24 | 9.4 | 3.78 | 24 |
| | Joint engagement | CSBCDP, shared positive affect | 2.42 | 2.93 | 3.54 | 3.56 | 4.96 | 5.48 | 24 | 7.57 | 9.76 | 24 |
| | Expressive language | MSEL, expressive language | 23.92 | 5.5 | 25.92 | 8.12 | 31.36 | 12.12 | 24 | 34.52 | 12.33 | 24 |
| | Developmental quotient | MSEL, nonverbal cognition | 27.5 | 8.27 | 31.12 | 9.86 | 30.28 | 16.62 | 24 | 34.44 | 16.67 | 24 |
| Lawton et al. (2012) | Initiating joint attention | ESCS: frequency of initiating joint attention] | 11.89 | 10.01 | 13.29 | 7.34 | 15.33 | 10.89 | 9 | 9 | 7.23 | 7 |
| | Joint engagement | Supported engagement | 4.27 | 1.77 | 5.58 | 0.81 | 5.58 | 0.81 | 9 | 4.11 | 1.68 | 7 |
| Pajareya et al. (2011) | Developmental quotient | FEDQ | 40.69 | 15.34 | 44 | 12.92 | 44 | 12.88 | 16 | 44.8 | 13.4 | 44 |
| | Autism symptoms: general | CARS | 39.69 | 6.59 | 35.25 | 6.08 | 37.18 | 6.22 | 16 | 34.5 | 6.01 | 16 |
| | Adaptive behavior | VABS, adaptive composite | 59.3 | | 64 | | 62.7 | 6.8 | 7 | 63.3 | 5.7 | 4 |
| Reitzel et al. (2013) | Joint attention responding | FBSA, response to name when sitting | 2 | | 2 | | 1.7 | 1.3 | 7 | 1.6 | 2.1 | 4 |
| | Communication | VABS, communication | 64.4 | 12.8 | 68.5 | 17 | 68.4 | 7.7 | 26 | 74.2 | 15.5 | 14 |
| Roberts et al. (2011) Home-based | Socialization | VABS, socialization | 68.7 | 7.3 | 70.8 | 9.9 | 66.4 | 7.7 | 27 | 73.1 | 10.8 | 14 |
| | Receptive language | RDLS, comprehension | 4.2 | 9.2 | 7.2 | 15.2 | 2.6 | 8.4 | 27 | 5.7 | 12.1 | 14 |
| | Expressive language | RDLS, expression | 3.4 | 8.3 | 6.0 | 10.9 | 2.8 | 7.5 | 26 | 4.4 | 8.7 | 14 |
| | Adaptive behavior | DBC | 44.7 | 19.0 | 43.9 | 21.9 | 52.9 | 29.3 | 22 | 42.9 | 24.3 | 13 |
| | Parental stress | PSInd. | 90.4 | 18.9 | 97.1 | 22.0 | 92.7 | 20.9 | 20 | 90.4 | 22.9 | 11 |
| | Communication | VABS, communication | 66.9 | 12.5 | 68.5 | 17.0 | 76.1 | 17.1 | 29 | 74.2 | 15.5 | 14 |
| Roberts et al. (2011) Center-based | Socialization | VABS, socialization | 70.1 | 7.3 | 70.8 | 9.9 | 72.6 | 11.2 | 29 | 73.1 | 10.8 | 14 |
| | Receptive language | RDLS, comprehension | 5.5 | 10.6 | 7.2 | 15.2 | 10.5 | 17.4 | 26 | 5.7 | 12.1 | 14 |
| | Expressive language | RDLS, expression | 8.2 | 16.6 | 6.0 | 10.9 | 7.0 | 15.1 | 26 | 4.4 | 8.7 | 14 |
| | Adaptive behavior | DBC | 58.5 | 20.4 | 43.9 | 21.9 | 55.7 | 19.5 | 22 | 42.9 | 24.3 | 13 |
| | Parental stress | PSInd. | 103.4 | 19.6 | 97.1 | 22.0 | 98.2 | 20.1 | 20 | 90.4 | 22.9 | 11 |
| | Autism symptoms: qualitative impairments in social | Modified ADOS: social affect | 29.5 | 9.2 | 34.1 | 8.7 | 26.6 | 10.1 | 49 | 27.3 | 10.6 | 49 |

| | | | | | | | | | | | | |
|---|---|---|---|---|---|---|---|---|---|---|---|---|
| Rogers et al. (2012) | Autism symptoms: restricted repetitve and stereotyped patterns of behavior, interests, and activities | ADOS, restrictive and repetitive | 3.92 | 2.01 | 4.31 | 1.92 | 3.96 | 1.86 | 49 | 3.82 | 2.04 | 49 |
| | Developmental quotient | MSEL, developmental quotient | 64.88 | 17.22 | 63.08 | 15.93 | 69.82 | 17.9 | 49 | 67.92 | 17.93 | 49 |
| | Receptive language | MCDI, phrases understood | 8.22 | 7.02 | 9.38 | 7.95 | 12.73 | 9.11 | 49 | 14.77 | 8.14 | 49 |
| | Expressive language | MCDI, vocabulary production | 12.24 | 35.6 | 12.44 | 39.72 | 42.27 | 61.99 | 49 | 38.87 | 73.71 | 49 |
| | Communication | VABS, communication | 67.66 | 13.19 | 67.29 | 11.05 | 72.55 | 12.06 | 49 | 74.29 | 14.55 | 49 |
| | Socialization | VABS, socialization | 76.68 | 8.74 | 77.95 | 8.01 | 77.32 | 9.19 | 49 | 78.67 | 10.78 | 49 |
| | Adaptive behavior | VABS, composite | 76.76 | 10.3 | 78.22 | 8.88 | 77.43 | 9.59 | 49 | 80.33 | 11.34 | 49 |
| | Imitation | Imitative sequences | 3.78 | 3.12 | 2.53 | 2.6 | 4.58 | 3.45 | 49 | 3.76 | 3.44 | 49 |
| | Responding to joint attention | Orient to joint attention | 0.35 | 0.35 | 0.28 | 0.33 | 0.34 | 0.29 | 49 | 0.34 | 0.34 | 49 |
| | Responding to joint attention | Observational, responding to initiating joint attention | 0.06 | 0.13 | 0.25 | 0.32 | 6.29 | 5.53 | 11 | 0.58 | 0.68 | 12 |
| | Initiating joint attention | Observational; initiating joint attention | 0.14 | 0.26 | 0.19 | 0.33 | 3.27 | 3.17 | 11 | 0.86 | 0.77 | 12 |
| | Communication | VABS, communication | 75.9 | 13.51 | 68.08 | 19.77 | 75.9 | 13.51 | 11 | 68.08 | 19.77 | 12 |
| | Receptive language | MSEL, receptive language | 28.27 | 11.35 | 25.33 | 8.52 | 28.27 | 11.35 | 11 | 25.33 | 8.52 | 12 |
| | Expressive language | MSEL, expressive language | 33.27 | 15.79 | 27.17 | 11.21 | 33.27 | 15.79 | 11 | 27.17 | 11.21 | 12 |
| | Maternal synchronization | Maternal synchronization | 0.57 | 0.03 | 0.63 | 0.03 | 0.72 | 0.04 | 34 | 0.61 | 0.04 | 30 |
| Siller et al. (2013) | Expressive language | MSEL, expressive language | 3.70 | 0.16 | 3.75 | 0.16 | 4.02 | 0.16 | 34 | 3.90 | 0.17 | 30 |
| | Joint engagement | mean of P/CSBS and ABS | 3.47 | 1.67 | 3.83 | 1.80 | 3.81 | 1.62 | 31 | 3.05 | 1.54 | 28 |
| | Developmental quotient | BSID, mental development index | 50.53 | 11.18 | 50.69 | 13.88 | 66.49 | 24.08 | 15 | 49.67 | 19.74 | 13 |
| Smith et al. (2000) | Receptive language | RDLS, comprehension | 13.47 | 3.6 | 13.69 | 3.73 | 42.87 | 22.29 | 15 | 33 | 16.86 | 13 |
| | Expressive language | RDLS, expressive | 15.13 | 0.52 | 16.31 | 2.69 | 44.53 | 23.48 | 15 | 36.23 | 21.19 | 13 |
| | Adaptive behavior | VABS, composite | 63.44 | 9.35 | 65.17 | 9.44 | 61.19 | 29.72 | 15 | 58.5 | 16.58 | 13 |
| | Stress thermometer ratings | Parenting stress | 2.51 | 0.82 | 2.06 | 0.87 | 1.86 | 0.88 | 35 | 2.26 | 1.09 | 35 |
| Tonge et al. (2006) | Developmental quotient | PEP-R, developmental quotient | 64.74 | 27.41 | 63.31 | 28.52 | 72.18 | 24.77 | 35 | 67.72 | 28.14 | 35 |
| Tonge et al. (2014) | Receptive language | RDLS, comprehension | 9.8 | 15.72 | 14.47 | 21.32 | 14.06 | 19.67 | 35 | 19.18 | 22.17 | 35 |
| Tonge et al. (2014) | Expressive language | RDLS, expression | 14.77 | 16.83 | 14.41 | 18.24 | 17.17 | 17.07 | 35 | 18.24 | 20.65 | 35 |
| | Autism symptoms: general | CARS | 41.23 | 5.25 | 38.97 | 5.79 | 35.86 | 6.14 | 35 | 38.73 | 6.73 | 35 |
| | Joint engagement | Communication acts | 0.29 | 0.49 | 2.00 | 2.24 | 9.71 | 14.08 | 7 | 1.86 | 2.67 | 7 |
| Venker et al. (2011) | Maternal synchronization | Parent redirecting behaviours | 14.0 | 8.6 | 12.3 | 10.0 | 4.3 | 3.4 | 7 | 14.3 | 15.4 | 7 |
| | Developmental quotient | MSEL, developmental quotient | 57.1 | 15.6 | 53.7 | 24.5 | 63.7 | 17.4 | 10 | 58.1 | 25.0 | 10 |
| Welterlin et al. (2012) | Parental stress | PSInd., total score | 254.1 | 48.5 | 244.5 | 32.8 | 242.4 | 41.5 | 10 | 256.2 | 70 | 10 |
| | Joint engagement | SIB-R, social interaction | 11.8 | 6.1 | 12.1 | 4.2 | 18.4 | 7.3 | 10 | 16 | 5 | 10 |
| | Receptive language | SIB-R, language comprehension | 9.9 | 4.4 | 7.3 | 4.2 | 12.0 | 4.7 | 10 | 10.9 | 4.8 | 10 |
| | Expressive language | SIB-R, language expression | 11.4 | 5.7 | 10.2 | 7.5 | 16.2 | 7.1 | 10 | 14.2 | 7.1 | 10 |

ADOS means Autism Diasnostic Observation Schedule. MSEL means Mullen Scales of Early Learning. VABS means Vineland Adaptive Behavior Scale. ESCS means Early Social Communication Scale. PCFP means Parent-Child Free Play Procedur. PIA-CV means Parent Interview for Autism-Clinincal Version. CBRS means Child Behavior Rating Scale. RBS means Repetitive Behavior Scale. GSMD means Griffiths Scale of Mental Development. MCDI means MacArthur-Bates Communicative Developmental Inventory. ADI means Autism Diagnostic Interview. PSInv. means Parental Stress Inventory. RDLS means Reynell Developmental Language Scales. PLS means Preschool Language Scale IV. PSInd. means Parental Stress Index. SDQ means Strengths and Difficulties Questionnaire. IRS means Inteaction Rating Scale. UIA means Unstructured Imitation Assessment. SES means Social Emotion Scale. SCQ: C means Social Communication Questionnaire: Current Form. DAS means Differential Abilities Scale. CSBDP means Communication and Symbolic Behavior Scales Developmental Profile. CARS means Childhood Autism Rating Scale. FEDQ means Functional Emotional Developmental Questionnaire. FBSA means Functional Behavior Skills Assessment. DBC means Developmental Behaviour Checklist. BSID means Bayley Scales of Infant Development. PEP-R means Psychoeducational Profile-Revised. SIB-R means Scales of Independent Behavior-Revised.
