# Supplementary material for: Meta-analyses of individual versus group interventions for pre-school children with autism spectrum disorder (ASD)
Source: PLoS One. 2018 May 15;13(5):e0196272. doi: 10.1371/journal.pone.0196272 (PMC5953451; doi:10.1371/journal.pone.0196272)
Supplement: S3 Table — (PDF) [file pone.0196272.s006.pdf]

S3 Table. The results of Analysis II on each outcome

	Outcome	Individual p value	SMD (95%CI)	I^2(%)	Group p value	SMD (95%CI)	I^2(%)
Primary outcome	Autism general symptoms	p<0.01**	-0.30[-0.53, -0.88]	0	0.07	-0.44[-0.92, 0.03]	0
Secondary outcomes	Developmental quotient	0.02*	0.23[0.03, 0.42]	0	0.30	0.23[0.04, 0.41]	0
	Developmental quotient (baseline imbalance-adjusted)	0.06	0.20[-0.01, 0.40]	0	0.40	0.25[-0.32, 0.81]	N/A
	Expressive language	0.04*	0.17[0.01, 0.33]	0	0.61	0.07[-0.19, 0.33]	0
	Expressive language (baseline imbalance-adjusted)	0.02*	0.19[0.03, 0.36]	0	0.55	0.08[-0.27, 0.42]	0
	Receptive language	0.40	0.08[-0.11, 0.28]	14	0.79	-0.04[-0.34, 0.26]	2
	Reciprocity of social intercation towards others	p<0.001***	0.50[0.31, 0.69]	0	0.42	0.23[-0.33, 0.78]	53
	Adaptive behaviour	0.81	-0.02[-0.32, 0.16]	0	0.08	0.55[-0.06, 1.16]	0
Other outcomes	Qualitative impairment in social interaction	0.63	-0.07[-0.33, 0.20]	25	N/A		
	Qualitative impairment in communication	0.60	-0.07[-0.33, 0.19]	0	N/A		
	Restricted repetitive and stereotyped patterns of behaviour, interests, and activities	0.09	-0.20[-0.44, 0.03]	9	N/A		
	Initiating joint attention	0.06	0.42[-0.01, 0.85]	0	0.18	0.24[-0.11, 0.60]	0
	Responding to joint attention	0.12	0.56[-0.15, 1.27]	96	N/A		
	Imitation	0.18	0.54[-0.25, 1.33]	62	N/A		
	Parental synchrony	p<0.001***	0.98[0.30, 1.66]	84	N/A		
Estimates	Parenting stress	0.59	-0.13[-0.61, 0.34]	0	0.90	-0.10[-0.42, 0.21]	0

P-value indicates value of the test of overall synthesis. SMD indicates standard mean difference of the synthesised effect. 95% CI indicates 95% confidence interval of the SMD of the overall synthesis. *, **, and *** indicate statistically significant effects (p < 0.05, p < 0.01, and p < 0.001, respectively) in the analysis. N/A indicates data synthesis could not be performed due to lack of available studies. “baseline imbalance-adjusted” indicates results of the analysis after excluding the studies with baseline imbalances.
