# Supplementary material for: Meta-analyses of individual versus group interventions for pre-school children with autism spectrum disorder (ASD)
Source: PLoS One. 2018 May 15;13(5):e0196272. doi: 10.1371/journal.pone.0196272 (PMC5953451; doi:10.1371/journal.pone.0196272)
Supplement: S4 Table — (PDF) [file pone.0196272.s007.pdf]

S4 Table. The results of sensitivity analyses for Analysis I with cluster-robust variance estimation (Analysis IV)

	Individual intervention				Group intervention			
Outcome	Estimate	RSE	95%CI	p-value	Estimate	RSE	95%CI	p-value
Autism general symptoms	-0.30	0.16	[-0.69, 0.08]	0.10	N/A			
Developmental quotient	0.23	0.14	[-0.11, 0.56]	0.16	N/A			
Developmental quotient (baseline imbalance-adjusted)	0.20	0.14	[-0.13, 0.52]	0.19	N/A			
Expressive language	0.17	0.14	[-0.17, 0.51]	0.27	0.07	0.16	[-1.95, 2.09]	0.74
Expressive language (baseline imbalance-adjusted)	0.20	0.15	[-0.16, 0.55]	0.24	0.08	0.24	[-2.98, 3.13]	0.80
Receptive language	0.07	0.16	[-0.30, 0.44]	0.66	-0.04	0.31	[-4.02, 3.93]	0.91
Reciprocity of social ineteraction towards others	0.50	0.13	[0.20, 0.81]	0.006**	0.17	0.59	[-7.32, 7.67]	0.82
Adaptive behavior	-0.02	0.15	[-0.39, 0.34]	0.89	0.55	0.10	[-0.75, 1.85]	0.12
Qualitative impairment in social interaction	-0.08	0.17	[-0.48, 0.31]	0.64	N/A			
Qualitative impairment in communication	-0.07	0.07	[-0.24, 0.10]	0.37	N/A			
RRB	-0.20	0.18	[-0.64, 0.23]	0.30	N/A			
Initiating joint attention	0.37	0.32	[-0.40, 1.14]	0.30	0.24	0.39	[-4.76, 5.25]	0.30
Responding to joint attention	0.55	0.84	[-1.44, 2.54]	0.54	N/A			
Parental synchrony	1.06	0.42	[0.08, 2.05]	0.04*	N/A			
Parenting stress	-0.13	0.21	[-0.64, 0.38]	0.56	-0.08	0.62	[-7.98, 7.82]	0.56

Estimates indicate the estimated standard mean difference in the random effects model with cluster-robust variance estimation. RSE indicates robust standard error. CI indicates confidence interval. RRB indicates restricted repetitive and stereotyped patterns of behaviour, interests, and activities. "p-value" indicates p value of the tests of coefficients with cluster-robust variance estimation. N/A indicates data synthesis could not be performed due to lack of available studies. * and ** indicate statistically significant effects ($p < 0.05$ and $p < 0.01$, respectively) in the analysis. “baseline imbalance-adjusted” indicates results of the analysis after excluding the studies with baseline imbalances.
