# Supplementary material for: Meta-analyses of individual versus group interventions for pre-school children with autism spectrum disorder (ASD)
Source: PLoS One. 2018 May 15;13(5):e0196272. doi: 10.1371/journal.pone.0196272 (PMC5953451; doi:10.1371/journal.pone.0196272)
Supplement: S5 Table — (PDF) [file pone.0196272.s008.pdf]

S5 Table. Demographics of the included studies for individual and group interventions for Analysis III

Intervention type	Outcome	Number of trials	Number of intervention condition participants	Minimum participant number of intervention condition	Maximum participant number of intervention condition	Mean of intervention condition	Number of control condition participants	Minimum partiipant number of control condition	Maximum participant number of control condition	Mean of control condition
Individual	1.1 Autism general symptoms	3	115	14	77	38.33	112	14	75	37.33
Individual	2.1 Developmental quotient	4	104	15	49	26	104	13	49	26
Individual	2.1 Developmental quotient (§baseline adjustment)	4	104	15	49	26	104	13	49	26
Individual	2.2 Expressive language	7	202	11	77	28.86	196	10	75	28.00
Individual	2.2 Expressive language (§ baseline adjustment)	7	202	11	77	28.8571	196	10	75	28
Individual	2.3 Receptive language	7	202	11	77	28.86	196	10	75	28.00
Individual	2.4 Reciprocity of social ineteraction towards others	5	147	12	77	29.40	145	11	75	29.00
Individual	2.5 Adaptive behavior	7	208	4	77	29.71	203	4	75	29.00
Individual	3.1 Qualitative impairment in social interaction	2	126	49	77	63.00	124	49	75	62.00
Individual	3.2 Qualitative impairment in communication									
Individual	3.3 RRB	3	150	24	77	50.00	147	23	75	49.00
Individual	3.4 Initiating joint attention	4	107	14	49	26.75	107	13	49	26.75
Individual	3.5 Responding to joint attention	3	79	11	49	26.33	80	12	49	26.67
Individual	3.6 Parental synchrony	3	101	10	77	33.67	98	9	75	32.67
Individual	3.7 Parenting stress	2	20	10	10	10.00	20	10	10	10.00
Group	1.1 Autism general symptoms									
Group	2.1 Developmental quotient									
Group	2.1 Developmental quotient (§baseline adjustment)									
Group	2.2 Expressive language									
Group	2.2 Expressive language (§ baseline adjustment)									
Group	2.3 Receptive language									
Group	2.4 Reciprocity of social ineteraction towards others	3	48	5	34	16.00	40	6	27	13.33
Group	2.5 Adaptive behavior									
Group	3.1 Qualitative impairment in social interaction									
Group	3.2 Qualitative impairment in communication									

Group	3.3	RRB									
Group	3.4	Initiating joint attention	2	43	9	34	21.50	34	7	27	17.00
Group	3.5	Responding to joint attention									
Group	3.6	Parental synchrony									
Group	3.7	Parenting stress	2	40	5	35	20.00	24	6	18	12.00
