# Supplementary material for: Meta-analyses of individual versus group interventions for pre-school children with autism spectrum disorder (ASD)
Source: PLoS One. 2018 May 15;13(5):e0196272. doi: 10.1371/journal.pone.0196272 (PMC5953451; doi:10.1371/journal.pone.0196272)
Supplement: S6 Table — (PDF) [file pone.0196272.s009.pdf]

S6 Table. Demographics of the included studies for individual and group interventions for Analysis IV

Intervention type	Outcome		Number of trials	Number of intervention condition participants	Minimum participant number of intervention condition	Maximum participant number of intervention condition	Mean of intervention condition	Number of control condition participants	Minimum partiipant number of control condition	Maximum participant number of control condition	Mean of control condition
Individual	1.1	Autism general symptoms	6	159	12	77	26.50	159	12	75	26.50
Individual	2.1	Developmental quotient	9	202	10	49	22.44	202	10	49	22.44
Individual	2.1	Developmental quotient (§baseline adjustment)	8	190	10	49	23.75	190	10	49	23.75
Individual	2.2	Expressive language	12	305	10	77	25.42	287	10	75	23.92
Individual	2.2	Expressive language (§ baseline adjustment)	11	279	10	77	25.36	273	10	75	24.82
Individual	2.3	Receptive language	11	275	10	77	25.00	257	10	75	23.36
Individual	2.4	Reciprocity of social ineteraction towards others	10	215	5	77	21.50	211	5	75	21.10
Individual	2.5	Adaptive behavior	8	230	4	77	28.75	216	4	75	27.00
Individual	3.1	Qualitative impairment in social interaction	10	215	5	77	21.50	211	5	75	21.10
Individual	3.2	Qualitative impairment in communication	N/A								
Individual	3.3	RRB	4	162	12	77	40.50	159	12	75	39.75
Individual	3.4	Initiating joint attention	7	147	5	49	21.00	150	6	49	21.43
Individual	3.5	Responding to joint attention	4	86	7	49	21.50	87	7	49	21.75
Individual	3.6	Parental synchrony	6	165	7	77	27.50	159	7	75	26.50
Individual	3.7	Parenting stress	3	40	10	20	13.33	31	10	11	10.33
Group	1.1	Autism general symptoms	N/A								
Group	2.1	Developmental quotient	N/A								
Group	2.1	Developmental quotient (§baseline adjustment)	N/A								
Group	2.2	Expressive language	N/A								
Group	2.2	Expressive language (§ baseline adjustment)	5	122	5	35	24.40	106	6	35	21.20
Group	2.3	Receptive language	4	98	5	35	24.50	82	6	35	20.50
Group	2.4	Reciprocity of social ineteraction towards others	4	72	5	34	18.00	64	6	27	16.00
Group	2.5	Adaptive behavior	2	27	5	22	13.50	19	6	13	9.50
Group	3.1	Qualitative impairment in social interaction	N/A								
Group	3.2	Qualitative impairment in communication	N/A								

Group	3.3	RRB	N/A								
Group	3.4	Initiating joint attention	3	67	9	34	22.33	58	7	27	19.33
Group	3.5	Responding to joint attention	N/A								
Group	3.6	Parental synchrony	N/A								
Group	3.7	Parenting stress	3	60	5	35	20.00	35	6	18	11.67
